# *SoxY* gene family expansion underpins adaptation to diverse hosts and environments in symbiotic sulfide oxidizers

**DOI:** 10.1128/msystems.01135-23

**Published:** 2024-05-15

**Authors:** Marta Sudo, Jay Osvatic, John D. Taylor, Suzanne C. Dufour, Anchana Prathep, Laetitia G. E. Wilkins, Thomas Rattei, Benedict Yuen, Jillian M. Petersen

**Affiliations:** 1 University of Vienna, Centre for Microbiology and Environmental Systems Science, Vienna, Austria; 2 Doctoral School in Microbiology and Environmental Science, University of Vienna, Vienna, Austria; 3 Joint Microbiome Facility of the Medical University of Vienna and the University of Vienna, Vienna, Austria; 4 Department of Laboratory Medicine, Medical University of Vienna, Vienna, Austria; 5 Life Sciences, The Natural History Museum, London, United Kingdom; 6 Department of Biology, Memorial University of Newfoundland, St. John’s, Newfoundland, Canada; 7 Department of Biology, Faculty of Science, Prince of Songkla University, HatYai, Thailand; 8 Eco-Evolutionary Interactions Group, Max Planck Institute for Marine Microbiology, Bremen, Germany; University of Florida, Gainesville, Florida, USA

**Keywords:** sulfur metabolism, symbiosis, phylogenetic analysis, sulfur oxidation, microbial ecology, metagenomics, gene family expansion

## Abstract

Sulfur-oxidizing bacteria (SOB) have developed distinct ecological strategies to obtain reduced sulfur compounds for growth. These range from specialists that can only use a limited range of reduced sulfur compounds to generalists that can use many different forms as electron donors. Forming intimate symbioses with animal hosts is another highly successful ecological strategy for SOB, as animals, through their behavior and physiology, can enable access to sulfur compounds. Symbioses have evolved multiple times in a range of animal hosts and from several lineages of SOB. They have successfully colonized a wide range of habitats, from seagrass beds to hydrothermal vents, with varying availability of symbiont energy sources. Our extensive analyses of sulfur transformation pathways in 234 genomes of symbiotic and free-living SOB revealed widespread conservation in metabolic pathways for sulfur oxidation in symbionts from different host species and environments, raising the question of how they have adapted to such a wide range of distinct habitats. We discovered a gene family expansion of *soxY* in these genomes, with up to five distinct copies per genome. Symbionts harboring only the “canonical” *soxY* were typically ecological “specialists” that are associated with specific host subfamilies or environments (e.g., hydrothermal vents, mangroves). Conversely, symbionts with multiple divergent *soxY* genes formed versatile associations across diverse hosts in various marine environments. We hypothesize that expansion and diversification of the *soxY* gene family could be one genomic mechanism supporting the metabolic flexibility of symbiotic SOB enabling them and their hosts to thrive in a range of different and dynamic environments.

**IMPORTANCE:**

Sulfur metabolism is thought to be one of the most ancient mechanisms for energy generation in microorganisms. A diverse range of microorganisms today rely on sulfur oxidation for their metabolism. They can be free-living, or they can live in symbiosis with animal hosts, where they power entire ecosystems in the absence of light, such as in the deep sea. In the millions of years since they evolved, sulfur-oxidizing bacteria have adopted several highly successful strategies; some are ecological “specialists,” and some are “generalists,” but which genetic features underpin these ecological strategies are not well understood. We discovered a gene family that has become expanded in those species that also seem to be “generalists,” revealing that duplication, repurposing, and reshuffling existing genes can be a powerful mechanism driving ecological lifestyle shifts.

## INTRODUCTION

Sulfur oxidation is an ancient metabolic process with a rich evolutionary history. It involves numerous enzymatic pathways that have evolved to carry out distinct steps of sulfur oxidation from the most reduced, hydrogen sulfide, to the most oxidized, sulfate (1–3). Sulfur-oxidizing bacteria (SOB), which use sulfur oxidation to generate energy, are remarkably diverse and are widespread in nature (1, 2). Although they can be found worldwide, their simultaneous reliance on reduced sulfur compounds and oxidants such as oxygen means that they are restricted to the limited habitats where both co-occur. Presumably, competition among diverse SOB for the limited environments they can thrive in is intense. SOB have evolved a range of successful strategies to access oxidants and reductants, attuned to various microniches within these environments. These range from the remarkable morphology and migration behavior of some giant sulfur bacteria such as *Beggiatoa* sp. (4), to anaerobic growth on electron acceptors other than oxygen, such as in *Thioalkalivibrio denitrificans* that uses nitrate (5). In addition, many bacteria can transform sulfur compounds without relying exclusively on these for energy, such as *Roseobacter* spp. (6). This could explain why most environments ideal for sulfur oxidation host diverse communities of sulfur oxidizers and other microorganisms.

Evolving symbiosis with an animal host is also a highly successful way for SOB to access substrates. In symbiosis, SOB are thought to have continuous and simultaneous access to oxygen and reduced sulfur compounds that might otherwise be in limited supply or subject to intense competition among free-living bacteria in the external environment (7–9). In return, the symbionts use the energy they gain from sulfur oxidation to convert inorganic carbon into nutrients for the host (10). Symbioses with sulfur-oxidizing bacteria have evolved convergently on multiple occasions in at least seven eukaryotic phyla, including ciliates, tube worms, sponges, and lucinid clams (9, 11–14). Found in a variety of environments, from intertidal sediments to deep-sea hydrothermal vents, these symbioses face, at times, extreme and fluctuating concentrations of reduced sulfur compounds and electron acceptors in the environment (11). Additionally, biotic factors, such as symbiont localization within the host, mode of transmission, and fidelity, further shape these interactions (15). The range of microenvironments experienced by symbiotic SOB is therefore virtually as diverse as the hosts harboring them. The most diverse animal family that hosts symbiotic SOB is Lucinidae, which are marine bivalve clams (16). They are found in virtually all habitats where sulfur-oxidizing symbioses occur. Their symbionts are horizontally transmitted, taken up from the environment during host juvenile development (17). Once taken up, they are housed inside epithelial cells of the host gill (18). Considering that the symbionts must survive in the free-living environment, but also be adapted to an extremely intimate symbiosis with their host, lucinids represent an intersection of evolutionary forces, and therefore an excellent opportunity to understand genomic mechanisms of adaptation to hosts and environments.

Within the Gammaproteobacteria, symbiotic SOB comprise at least nine phylogenetically distinct clades (9, 19). All encode highly similar sulfur-oxidizing pathways in their genomes (12, 20, 21). The uniformity of sulfur metabolic pathways across diverse gammaproteobacterial symbiont lineages appears paradoxical in light of substantial variability in types and concentrations of sulfur compounds in their microenvironments, and the range of distinct pathways used by their free-living counterparts (9, 22, 23). However, the evolutionary forces shaping sulfur-oxidizing pathways within symbiotic SOB are not well understood.

The ability to adapt to novel habitats and shifting conditions can arise not only from the emergence of novel metabolic pathways but also from diversification of existing genetic elements (24). One prominent example in sulfur metabolism is seen in the genes encoding SoxY. SoxY is a sulfur anion carrier protein within the sulfur-oxidizing multienzyme complex (Sox pathway) (25, 26). The “canonical” SoxY has a highly conserved amino acid sequence in the sulfur-binding “swinging arm” region. In addition to the canonical *soxY*, the genomes of some SOB encode for divergent copies of the gene. Divergent SoxYs have mutations in the substrate-binding swinging arm region, potentially affecting substrate specificity (27–29). For example, mutations in the sulfur-binding region of SoxY in *Sulfurimonas denitrificans* enabled the oxidation of S8, a previously unrecognized Sox pathway substrate (30).

In this study, we investigated the evolution and diversification of the *soxY* gene family and its potential role in the diversification of SOB engaged in symbiotic associations with marine animals. We used comparative genomics and phylogenomic analyses to reveal sulfur-oxidation genes and pathways in 234 metagenome-assembled genomes (MAGs) of diverse marine symbiotic bacteria. Our findings indicate that although sulfur oxidation pathways are highly conserved across distinct phylogenetic clades of symbiotic SOB, there was a noteworthy expansion of the *soxY* gene family with some genomes containing as many as five distinct copies of the gene. Interestingly, symbionts that encode only the “canonical” *soxY* gene exhibited potential specialization for thiosulfate as a substrate and seem to be confined to a very few specialized hosts or environments. In contrast, symbionts with multiple divergent *soxY* genes form associations with diverse hosts across various environments. The expansion and diversification of the *soxY* gene family may have enabled symbionts and their hosts to effectively colonize a wider range of diverse environments.

## MATERIALS AND METHODS

### Sample collection

#### New collections

We collected clams from multiple sites and environments with detailed descriptions in Table S1. *Divalinga weberi n* = 1 and *Anodontia alba n* = 1 were collected in beds of *Thalassia testudinum* in Bocas del Toro, Panama. Both clams were sampled by snorkeling and digging sediment, then the clams were sieved out and stored in RNAlater at −20°C. *Euanodontia ovum n* = 1 was collected from Lazarus beach on St John’s Island, Singapore. The clam was collected by sieving sediment dug up from a seagrass bed during low tide. Further individuals including *Loripes orbiculatus n* = 11, *Loripinus fragilis n* = 7, and *Lucinella divaricata n* = 5 were collected by sieving sediment dug up from seagrass (*Cymodocea nodosa*) meadows in Piran, Slovenia and fixed immediately in RNAlater (Cat. No. AM7020; Life Technologies, USA) at 4°C overnight and stored at −20°C until extraction. *Rugalucina vietnamica n* = 5 was collected from an intertidal seagrass bed using a shovel at approx. 10 cm depths. Sediment was sieved through a 1-mm mesh, and bivalves were collected. *Thyasira* cf. *gouldii* specimens were sieved (1 mm mesh) out of sediment collected at roughly 15 m water depth using a Petersen grab at Neddy’s Harbour, Bonne Bay, Newfoundland, Canada. The *T*. cf. *gouldii* specimens were transported to Memorial University, St. John’s, Newfoundland, where the gills were dissected, preserved in RNAlater, and stored at −20°C until extraction.

#### Museum samples

We carefully dissected gill tissue fragments using clean scalpels and forceps from lucinid clam samples (Table S1) at the Natural History Museum in London, UK, and the Florida Museum of Natural History, Gainesville, FL, USA. Access to the Florida Museum collection was graciously provided by Gustav Paulay and Amanda Bemis. All museum collection samples were preserved in 70%–100% ethanol and stored at 13°C–25°C.

### DNA extraction and library preparation for metagenome sequencing

DNA was extracted from gill tissue of 29 lucinid and 1 thyasirid clam species from diverse vegetated and unvegetated environments (Table S1). Information such as habitat type, precise location, and depth were recorded when available and are found in Table S1. The DNA was extracted from approximately 1 cm^2^ gill fragments using three different extraction methods: the Qiagen DNeasy Blood and Tissue Kit (Cat. No. 69506; Qiagen, USA), the animal tissue protocol from the Analytik Jena Innuprep DNA Mini Kit (Cat. No. 845-KS-1041250, Germany), and phenol-chloroform method. Detailed protocols are described in the Supplemental Text.

Sequencing library preparation and metagenomic and amplicon sequencing were carried out by the Joint Microbiome Facility of the Medical University of Vienna and the University of Vienna. Libraries were constructed using Illumina compatible library prep kits (NEBNext Ultra II FS DNA Library Prep Kit, NEBNext Ultra DNA v2 Library Prep Kit, and Nextera Mate Pair Sample Preparation Kit). All libraries were sequenced with Illumina technology (HiSeq 3000, HiSeq 4000, and NovaSeq 6000) using paired-end settings with read lengths of 100, 150, or 250  bp to generate a minimum of 1,000,000 reads (see Table S1 for more details).

### Quality filtering, assembly, and bacterial genome binning

Quality filtering, assembly, and bacterial genome binning were done according to the workflow used in reference 31. Read libraries were trimmed, contamination filtered, and quality checked using BBMap v37.61’s BBDUK (32) feature and the software’s adapter and PhiX databases to detect contaminants. Libraries were interleaved as needed for future processing and analysis. Individual read libraries were assembled using SPAdes v3.13.1 (33, 34). Read libraries were mapped to the scaffold assemblies using BBMap with default settings. The resulting sam files were converted to bam files with samtools v1.9 (35) and sorted using the anvi-init-bam script from Anvi’o (36). The assembled scaffolds were then binned using a combination of Anvi’o v6.1 or 7.1 using CONCOCT v1.1.0 (37), and metaBAT v2.15 (38). All potential bins for each metagenome were then compared using dRep v2.4.2’s (39) dereplicate workflow with default settings, and the one considered the best was selected automatically. If no bin was selected, a manual bin selection (using CheckM v1.1.3 [40] with Gammaproteobacteria gene set) and revision of the best metagenome-assembled genome using Anvi’o’s “anvi-refine” were attempted to manually improve the quality of the MAGs. Scripts for this workflow can be found in the supplemental material from reference 22.

Further MAGs of symbionts of lucinids and other marine invertebrates were retrieved from publicly available assemblies deposited in the NCBI database as well as MAGs of free-living bacteria *Allochromatium vinosum* (41), *Sedimenticola thiotaurini* (42), and *Sedimenticola selenatireducens* (43) (Table S2). To reduce the number of possible false negatives in gene searches, all symbiont genomes were analyzed for genome completeness and contamination using CheckM (40). The CheckM analysis was performed according to the default taxonomic-specific workflow using Gammaproteobacteria as the taxonomic rank. The minimum required completeness and contamination for SoxY analyses were above 90% and below 5%, respectively, with a few exceptions important for taxonomic diversity listed in Table S2 resulting in a total of 234 genomes. While interpreting the data from the MAGs of lower completeness during presence/absence analysis of sulfur genes, we considered that the absence may have been caused by lower completeness. Furthermore, two of thyasirid symbiont MAGs, belonging to the same species, showed substantial contamination (5.06%–6.65%); therefore, the interpretation of sulfur gene presence results for these MAGs was considered estimates.

### Annotation and analysis of sulfur oxidation metabolism

Putative sulfur genes from all symbiont genomes were identified using HMSS2 (44) run on default settings and using blastp (BLAST+ 2.14.0) (45) search using known identified sulfur proteins from *A. vinosum* as a query with a maximal *e*-value 10e−30 and 10e−6 for short proteins under 120 amino acids (Table S3).

### Phylogenomic analyses of *soxY* genes in symbiotic and free-living SOB

#### Data collection

To identify *soxY* genes, all MAGs and genome assemblies were first annotated using Prodigal v2.6.3 using default settings (46), and the predicted protein sequences queried with the SoxY HMM model obtained from the eggNOG database v5.0 (47) (accessed January 2022) and using the hmmsearch tool in HMMER v3.3.2 (48) with an *e*-value cutoff of 10^−5^ (based on the observed sequence identity of SoxY and SoxZ proteins). To reconstruct the SoxY phylogeny, sequences from a reference proteomes alignment of SoxY (PF13501) covering 55% of SoxY sequences (RP55) in representative proteome groups containing similar proteomes were calculated based on co-membership in UniRef50 clusters resulting in 1,673 sequences (obtained on 30 August 2022). Moreover, 44 viral SoxY sequences (49) were also included by search of MAGs as described for symbiont sequences. The final list of putative SoxY sequences was created by combining the SoxYs from symbiotic and viral MAGs and RP55 SoxY sequences and further verification for the presence of SoxY (PF13501) functional domain using the InterProScan (50) search on default settings. To optimize the usage of computational resources, the number of total sequences was reduced from 2,291 to 1,960 by the deletion of identical proteins using the deduplicate sequences function (rmdup) in seqkit v2.2.0 (51). Sequences of poor quality (less than 140 aa length, no “GGC'' functional motif) were deleted resulting in a total of 1,631 sequences.

#### Phylogenetic analysis of SoxY sequences

The final set of full-length SoxY sequences was aligned using hmmalign (48) (default settings), and the SoxY model retrieved from eggNOG database (47). The alignment was visualized using MEGA X (52), and poorly aligned regions were manually identified and trimmed. During manual inspection of the alignment, a further 31 low-quality sequences were removed resulting in a final number of 1,631 sequences. A maximum likelihood tree was constructed using IQ-Tree multicore version 2.1.2 (53, 54) with the Q.pfam+I+G4 model (ModelFinder [55] was used to determine the best substitution model) and 10,000 ultra-rapid bootstraps (UFBoot) (56). The final consensus tree was visualized and annotated using Interactive Tree Of Life (iTOL) v6.8 (57) as well as Inkscape 1.3.2. To establish if *soxY* and *soxZ* genes, encoding for two functionally connected dimers of SoxYZ proteins, coevolved, the phylogenetic of SoxZ sequences was performed using SoxZ sequences associated with analyzed SoxY sequences (detailed description in Supplemental Text).

### Phylogenomic reconstruction of the lucinid symbiont relationships

Newly obtained symbiotic MAGs as well as publicly available MAGs of previously described lucinid symbionts (*Ca*. Thiodiazotropha sp., *Ca*. Sedimenticola sp., and Thiohalomonadales sp.), alongside symbiotic bacteria of marine hosts and MAGs of free-living close relatives to *Ca*. Thiodiazotropha obtained from the NCBI database, were included in the analysis (NCBI accession numbers in Table S2). All publicly available MAGs we used were quality checked using CheckM’s taxonomy-specific workflow using the gammaproteobacterial set of marker genes (40). All MAGs were taxonomically classified using GTDB-Tk v0.3.3 classify workflow. This workflow produced a concatenated amino acid sequence alignment of 120 highly conserved single-copy bacterial marker genes that was used for phylogenomic analyses (46, 58–62). The alignment was constructed using IQ-Tree multicore version 2.1.2 (53, 54) using the default settings (auto substitution model detection—LG+I+G and 1,000 ultra-rapid bootstraps (UFBoot) (56). The tree was rooted at midpoint and visualized using iTOL v5 (57). The resulting phylogenetic tree was used alongside ANI analyzes to identify species-level clades. The average nucleotide identity (ANI) between MAGs was calculated using FastANI v1.3 (59), with a program-suggested 95% one-way ANI used as the threshold for species delimitation.

### Gene organization visualization in lucinid symbionts

The lucinid symbiont MAGs were annotated with the Rapid Annotation using Subsystem Technology (RAST) web server (63) using the RASTtk pipeline (64) and analyzed in SEED (65). The genes of interest were identified by the internal BLAST function in SEED. For each of the gene, models surrounding the *soxY* locus were annotated in RAST and verified using searches of the BLASTp against non-redundant protein sequences (nr) (66, 67), Pfam database (68), the eggNOG database (47) as well as InterProScan (50). The analysis was done on one representative MAG from each symbiont species (ANI above 95%) with the least number of contigs (Table S4). In case of substantial fragmentation of the analyzed region, a MAG with the second lowest fragmentation was chosen. For each of the tools, the search was performed on default settings with an *e*-value cutoff of 10^−5^. The gene visualization was based on the annotation overview in the SEED genome browser and illustrated using Inkscape 1.3.2.

## RESULTS

### Diverse symbiotic bacterial genomes revealed through metagenomic analysis of lucinid and thyasirid hosts

We sequenced, assembled, and binned the metagenomes of 68 lucinid and thyasirid individuals, representing 1 thyasirid species and 27 different lucinid host species from 4 subfamilies (Table S1). A total of 72 bacterial metagenome-assembled genomes were retrieved, out of which 60 were considered “high quality” because they were over 90% complete and less than 5% contaminated (Table S2) (69). All 72 MAGs were assigned to the Gammaproteobacteria class, specifically to the order Chromatiales and the family Sedimenticolaceae based on GTDB release 212 (Table S2). To expand the scope of our study, we included additional 162 publicly available MAGs of marine symbiotic bacteria associated with animal hosts (Table S2). Our analysis encompassed a total of 234 symbiotic bacterial genomes, spanning approximately 89 animal host species, from 5 phyla. The geographical scope encompassed 100 sites across 6 continents, representing diverse ecological contexts (Tables S1 and S2; Fig. S1).

**Fig 1:**
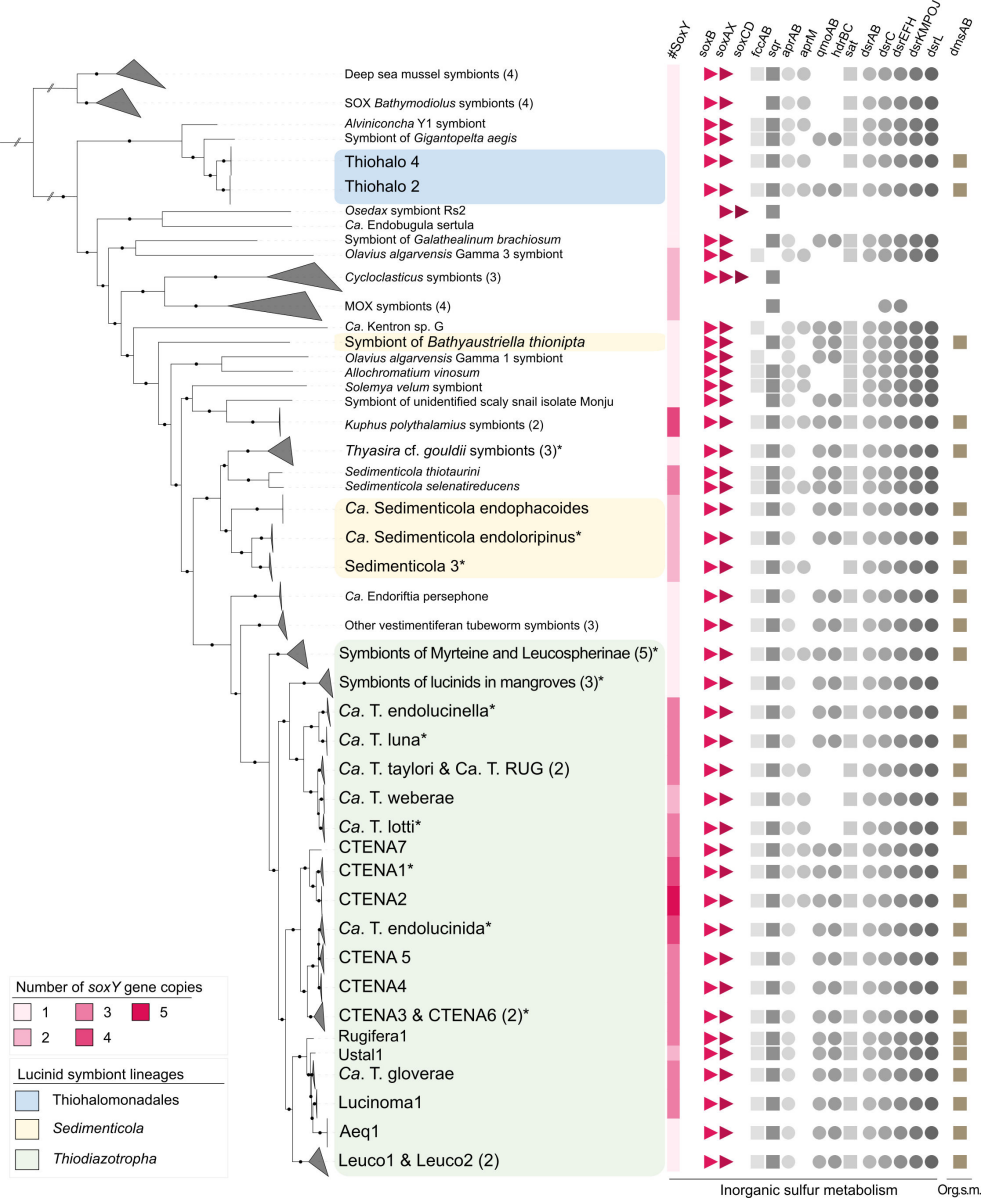
Maximum likelihood phylogenetic tree of symbiotic bacteria reconstructed from GTDB’s multisequence alignment using the best-fit model LG+I+G and 1,000 bootstraps. Black circles describe bootstrap support values above 85%. Clades containing symbiont MAGs generated in this study are indicated by asterisks. Number of symbiont species in each of the clades is indicated by the number in brackets. Lucinid symbiont genera are indicated in the following colors: Thiohalomonadales, blue; *Sedimenticola*, yellow; *Thiodiazotropha*, green. The first column describes the number of encoded *soxY* genes in each symbiont MAG. Further columns describe presence or absence of major sulfur-oxidizing genes within inorganic and organic (Org. s. m.) sulfur metabolism.

Through phylogenomic analyses employing a 95% average nucleotide identity threshold for species delimitation, 69 distinct bacterial species clades were identified among the symbiont MAGs (Fig. 1; Fig. S3; Table S5). This large data set allowed us to identify several novel symbionts. We retrieved a total of 47 novel *Thiodiazotropha* MAGs, comprising 14 symbiont species, 8 of which were previously undescribed, that were associated with 21 lucinid species from 3 different subfamilies (Fig. 1; Fig. S3; Table S5). We also identified 21 new MAGs representing 2 novel symbiont species from the genus *Sedimenticola* (*Ca*. Sedimenticola endoloripinus and Sedimenticola3), which were associated with 8 host species from the Pegophyseminae subfamily, marking the first comprehensive characterization of symbionts within this host subfamily (Fig. 1; Fig. S3; Table S5, Supplemental text). Finally, four MAGs, corresponding to three symbiont species, were retrieved from the *Thyasira* cf. *gouldii* (Fig. 1; Fig. S3; Table S5).

### Phylogenetically and ecologically diverse symbiotic SOB share highly conserved sulfur metabolisms

In contrast to highly diverse sulfur metabolic pathways known from free-living SOB, symbiotic SOB have been reported to have a highly conserved set of pathways; such uniformity is perhaps unexpected, given their phylogenetic diversity (12, 23). Our analysis across 69 different SOB symbiont species from multiple genera covering a great diversity of hosts and environments confirms earlier observations based on smaller data sets that sulfur oxidation metabolic pathways are either identical or of complementary function, meaning that the same reactions are carried out, but by different enzymes (Fig. 1; Fig. S3, Table S6). In symbiotic SOB, sulfur compounds are initially oxidized in the periplasmic space using the sulfide:quinone oxidoreductase (Sqr), sulfide dehydrogenase (FccAB), and the sulfur-oxidizing multi-enzyme (Sox) system (70) (Fig. 1; Fig. S2). Most symbiotic SOB encoded a truncated Sox pathway, lacking SoxCD, that accumulates polymeric water-insoluble sulfur globules within the periplasm as intermediate product (70, 71). They also shared the ability to subsequently oxidize these sulfur globules via the cytoplasmic dissimilatory sulfite reductase (Dsr) system, APS reductase, and ATP sulfurylase (Fig. 1; Fig. S2), which is advantageous when reduced sulfur compounds are not always available in the environment (70, 72, 73). The only genes that differed encoded the functionally complementary enzymes AprM and QmoAB/HdrBC (Fig. 1; Fig. S2). This toolkit for sulfur oxidation not only exhibited similarities in gene copy numbers but also shared relatively high amino acid identity across ecologically diverse symbiotic SOB genera originating from a wide range of environments (Fig. 5; Table S1). The gene encoding the sulfur oxidation protein SoxY was the sole exception, with an average amino acid identity of just 20% compared to the standard SoxY in *A. vinosum* and varying in copy numbers of up to five per genome (Fig. 1).

### Phylogenetic analysis of SoxY proteins reveals diversification into four major clades

**Fig 2:**
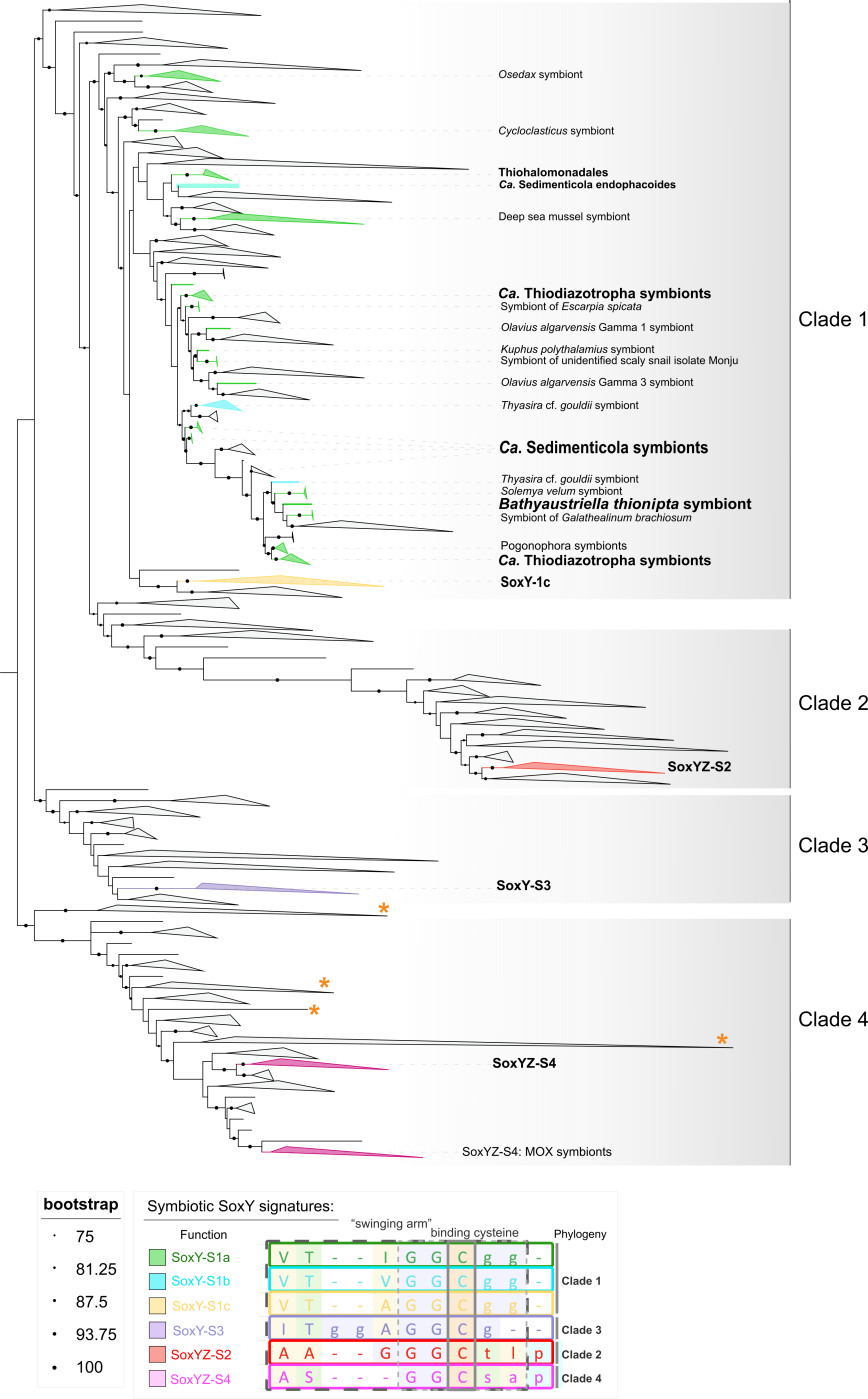
Maximum likelihood tree of SoxY proteins (1,631 sequences total) from symbionts and free-living bacteria including sequences from NCBI and Pfam (RP55). The major clades are labeled according to their phylogeny. The SoxY sequence signature groups from symbiotic SOB were distinguished by mutations within the sulfur-binding “swinging arm” of the protein (Fig. S6). The canonical SoxY sequence with its conserved sulfur-binding cysteine-110 (cys110) is labeled as SoxY-S1a. Presence of viral sequences within collapsed clades has been marked in clades 3 and 4 (orange star). Coloring according to the previous tree and legend. Branches with support over 75% (10,000 bootstraps) have been marked with black circles, the size of the circle is equivalent to the % support according to the legend.

We reconstructed the phylogenetic relationships of SoxY proteins from symbiotic and free-living SOB to investigate the evolutionary history of this gene family. The final set of 1,634 SoxY sequences, representing 247 symbiotic and 1,387 free-living bacteria and viruses, formed four major clades (Fig. S7). Clades 1 and 3 contained canonical thiosulfate-oxidizing SoxY proteins, characterized by the conserved “VTIGGC”' motif, previously established in *A. vinosum* (74) and *Paracoccus pantotrophus* (26, 75). Clade 1 was dominated by proteins from Gammaproteobacteria, including all examined symbiotic SOB, while clade 3 primarily consisted of SoxY proteins from metabolically versatile Alphaproteobacteria capable of chemolithoautotrophy and chemoorganoheterotrophy such as *Magnetospira* sp. (strain QH-2), *Methylosinus* sp. 3 S-1, and *Hyphomicrobium* spp. (Fig. S4). Clade 3 contained proteins from only a few symbiotic SOB. These were the symbionts of *Ca*. T. endolucinida*, Ca*. T. CTENA1, and *Ca*. T. CTENA2 (Fig. 2).

Clades 2 and 4, which comprised genes originating from a fusion of the SoxY and SoxZ subunits, were highly divergent, as evidenced by long branch lengths and low amino acid identity to the canonical SoxY sequence from the model sulfur oxidizer *Allochromatium vinosum* (Fig. 2; approximately 22%). The genes for SoxYZ sequences from clades 2 and 4 were present in the genomes of 15 *Ca*. Thiodiazotropha symbiont species and the symbiont of the shipworm *Kuphus polythalamius*. Furthermore, clade 2 additionally contained SoxYZ sequences from the lucinid symbiont *Ca*. T. lotti and from *Cycloclasticus* spp., which are known for short-chain alkane oxidation (76). Clades 2 and 4 also contained representatives from phylogenetically diverse and metabolically versatile free-living bacteria (clade 2: Alpha-, Beta-, Gammaproteobacteria and Campylobacterota; clade 4: Alpha-, Beta-, Delta-, and Gammaproteobacteria; Fig. S4) as well as viruses (clade 4; Fig. 2). These bacteria included *Thauera, Azoarcus,* and *Hyphomicrobium* species, which are mixotrophs or obligate heterotrophs and oxidize thiosulfate as an accessory metabolism (77, 78). Clade 4 also included SoxYZ sequences from the methane-oxidizing symbionts of *Bathymodiolus* mussels as well as *Methylococcus capsulatus* (79), *H. sulfonivorans* (80), *Methylocella silvestris* (81), all of which are not known to be capable of energy generation from thiosulfate oxidation.

### Six unique sequence signatures in the sulfur-binding “swinging-arm” region of SoxY proteins

Aside from segregating into four distinct clades, the SoxY sequences of symbiotic SOB had an additional feature: six distinct sequence signatures within the “swinging arm” region of the protein, which is the substrate-binding site (Fig. 2; Fig. S6). These signatures were polyphyletic traits that did not necessarily correspond to unique monophyletic SoxY clades (Fig. 2). Three of the signatures (1a, 1b, and 1c) were unique to clade 1 and were observed in both symbiotic and free-living Gammaproteobacteria (Fig. 2). Signature 1a, in particular, featured the highly conserved “VTIGGC” motif, a hallmark of the canonical thiosulfate-oxidizing SoxY described in model systems such as *A. vinosum* and *P. pantotrophus*, as well as relatively high amino acid sequence similarity to these model systems (*A. vinosum* between 52.2% and 64.2%, *P. pantotrophus* between 40% and 46.9%). Signatures 1b and 1c were distinguished from *1*a by only a single amino acid change (Fig. 2). Signature 1b was present in only four symbiont species: Three *Thyasira cf. gouldii* symbiont species, where it is the only *soxY* copy in the genome, and in *Ca*. Sedimenticola endophacoides, which also has a clade 1 *soxY* gene with the highly conserved signature *1a* (Fig. 2, cyan). Signature 1c was unique to a divergent group of genes from *Ca*. T. weberae and *Ca*. T. lotti with lower amino acid identity to the canonical *A. vinosum* SoxY (between 33% and 35%). Sister to this clade was a less divergent cluster of two sequences from *Ca*. T. CTENA2 symbiont species (exhibiting 43.5% amino acid similarity to *A. vinosum*). Signatures 2–4 were each observed in proteins from the clades of the corresponding name (clades 2–4; see Fig. 2; Fig. S7). Signatures 2–4 were highly divergent from the canonical SoxY of *A. vinosum*, with low amino acid identity (24%–33.3% for signature 2, 28.8%–31.6% for signature 3, and 26%–36.4% for signature 4) and multiple mutations within the “swinging arm” motif. Signature 3 was unique to lucinid symbionts within clade 3, while signature 2 (186 sequences) and signature 4 (297 sequences) were widely distributed among free-living bacteria throughout their respective clades. The SoxY variants will hereafter be referred to as SoxY-S1a, SoxY-S1b, SoxY-S1c, SoxYZ-S2, SoxY-S3, and SoxYZ-S4, where the suffix of each name denotes both the clade to which the amino acid sequence belongs, and the sequence signature pattern observed in the sulfur-binding region.

### Lucinid symbiont genomes reveal distinct genomic context and functional variations of SoxY signatures compared to free-living sulfur oxidizers

**Fig 3:**
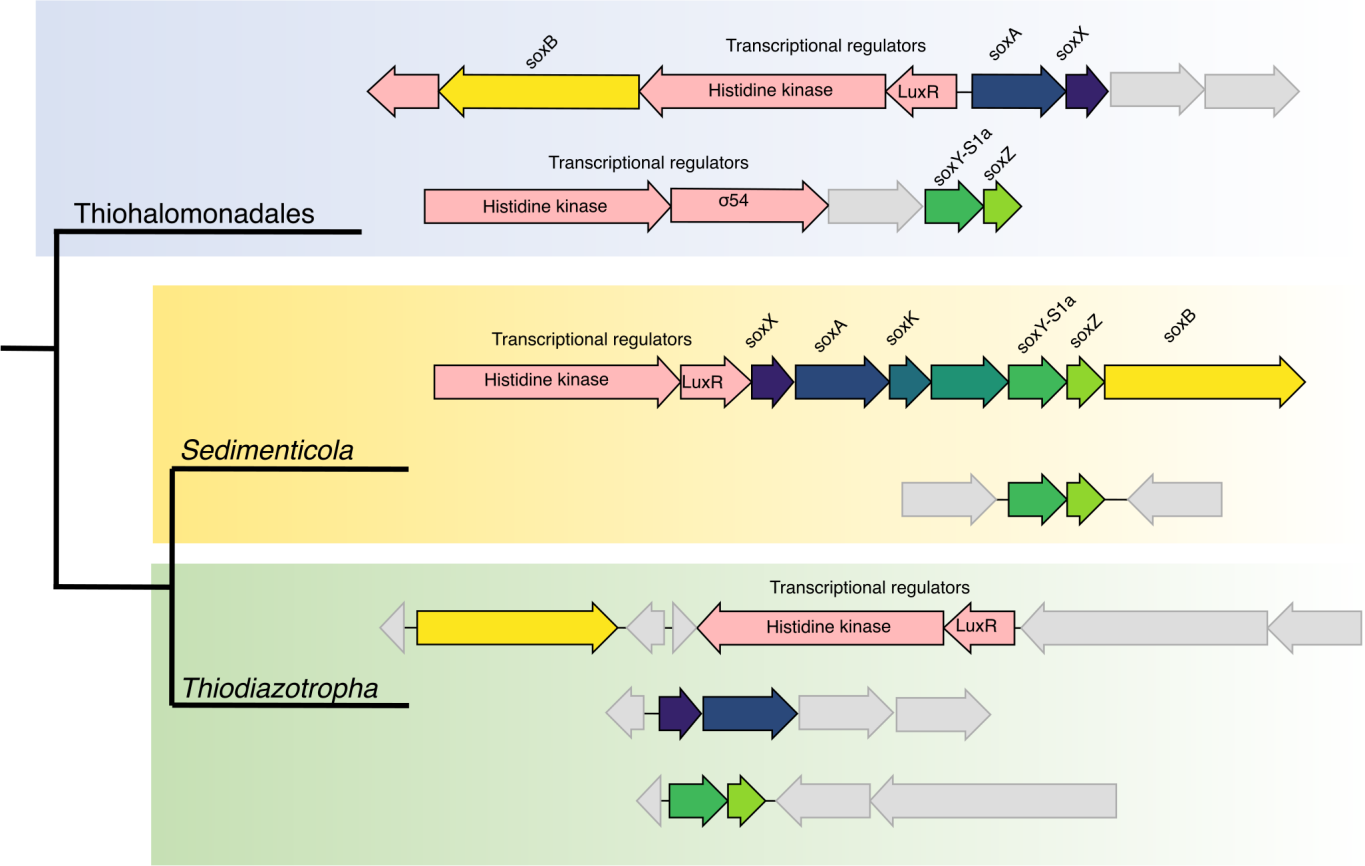
Genomic islands for sulfur oxidation across lucinid symbiont genera. SoxY displayed here is the canonical *soxY* signature 1a gene. All regulatory genes are marked in red. Hypothetical genes are marked in gray. Colors used for the *sox* genes in Thiohalomonadales and *Thiodiazotropha* correspond to the labeling in the *Sedimenticola* clade.

To gain insight into the potential roles of the different SoxY variants, we investigated the genome architecture and context of the Sox pathway components in lucinid symbiont MAGs from the Thiohalomonadales, *Sedimenticola,* and *Thiodiazotropha* groups. For SoxY-S1a, three main genomic organizational patterns emerged, which were consistent with symbiont phylogeny: (i) The Thiohalomonadales Sox pathway was split into two loci: one harboring *soxB* and *soxAX*, and the other containing the *soxYZ* genes (Fig. 3). The *soxB* and *soxAX* locus included genes encoding LuxR and histidine kinase genes, known transcriptional regulators in quorum sensing (82). Meanwhile, the *soxYZ* locus housed genes encoding the transcriptional regulators σ-54 and histidine kinase (Fig. 3) (ii). *Sedimenticola* had a *soxABXYZ* operon, situated downstream of the transcriptional regulators, the LuxR-encoding gene, and a histidine kinase (Fig. 3). Additionally, the *Sedimenticola* MAGs encoded a second copy of *soxY-S1a* and the adjacent *soxZ* in a separate genomic location, but no transcriptional regulators could be identified (iii). In *Thiodiazotropha*, the sox genes were segregated into three clusters, *soxB*, *soxAX*, and *soxYZ*, that were distributed across separate locations (Fig. 3). Only the *soxB* gene cluster included transcriptional regulators, LuxR protein, and a histidine kinase. No transcriptional regulators could be identified in the remaining gene clusters.

A further *soxY* gene variant from clade 1 is *soxY-S1b*, which we only found in the genus *Sedimenticola*, neighbored genes encoding beta-barrel assembly-enhancing proteases, ribosomal RNA small subunit methyltransferase E, and a chemotaxis methyl-accepting receptor (Fig. S5). Genes encoding the most divergent SoxY homologs in clade 1, *soxY-S1c*, were co-localized with genes involved in sulfur oxidation, such as the sulfur carrier protein *YeeE/D* and dissimilatory sulfate reductases *dsrAB*. Further upstream of the *soxY-S1c* was genes annotated as dimethyl sulfoxide reductases (*dmsAB*) that reduce DMSO to DMS. The lucinid *soxYZ-S2* was situated on a locus containing a PQQ-dependent quino(hemo)protein alcohol dehydrogenase (*ADH*), which oxidizes a variety of alcohols and sugars (83), and *soxH*, a sox gene of unknown function. Genes encoding SoxY-S3 were located upstream of a copy of *soxZ* and downstream of a gene encoding octaheme tetrathionate reductase (*otr*), which can reduce tetrathionate to thiosulfate but may also have a role in reducing nitrite (84, 85). Finally, genes for SoxYZ-S4 were clustered with those for formate (*fdhAB*) and methanol dehydrogenases (*xoxF*). It is noteworthy that the *soxYZ-S2* and *soxYZ-S4* fusion genes were also encoded with conserved synteny in the MAGs of the *Kuphus polythalamius* symbiont (*soxYZ-S2* and *soxYZ-S4*) and *Bathymodiolus* methane-oxidizing symbionts (*soxYZ-S4* only). The genome architectures described above were conserved across all the representative MAGs that were analyzed (Table S4).

## DISCUSSION

Partnering with animal hosts helps chemoautotrophic SOB avoid competition and provides an advantage over the free-living lifestyle by ensuring a constant supply of oxygen and reduced sulfur compounds. It is intriguing that although free-living SOB encode diverse pathways for sulfur oxidation, the pathways encoded by symbiotic SOB are largely conserved, although they stem from several distinct phylogenetic groups. This suggests that this set of sulfur oxidation mechanisms may provide a selective advantage to SOB in symbiosis with animal hosts.

### The central role of canonical *Sox* genes and thiosulfate oxidation in shaping symbiotic relationships

In this study, we characterized the *sox* genes, particularly the *soxY* gene family, from symbiotic systems to shed light on factors shaping their evolution. All SOB symbionts, except those associated with *Thyasira cf. gouldii,* possessed the clade 1 SoxY sequences with the S1a signature in the sulfur-binding region. The near-ubiquitous conservation of this gene and key residues within the binding region, which is the characteristic of the canonical SoxY, suggests these SOB symbiont SoxYs play a highly conserved role in binding thiosulfate. The conservation of this function is consistent with observations from physiological studies of these systems. For example, thiosulfate accumulates in the hemolymph of the lucinid *Stewardia floridana* when the clams are incubated with sulfide (86). Furthermore, the symbionts of *Bathymodiolus thermophilus* show a preference for thiosulfate (87). Members of genus *Sedimenticola* encode two copies of the canonical *soxY,* both with the S1a signature. Encoding two nearly identical canonical *soxY-S1a* variants could potentially increase SoxY activity through higher dosage as a result of gene duplication-amplification, possibly indicating specialization toward thiosulfate oxidation in this genus (88–90).

The organization of *soxABYZX* genes and their regulators into a single operon, as we saw in *Sedimenticola*, was previously observed in free-living SOB such as *P. pantotrophus*, *Chlorobium tepidum*, *Bradyrhizobium diazoefficiens*, and *A. thiooxidans* (91–93). An operon structure would enable co-regulation and co-transcription of the entire Sox pathway, minimizing stochastic variations in gene expression and reducing the regulatory information required for optimizing gene expression patterns (94, 95). In contrast to *Sedimenticola*, *sox* genes were dispersed throughout the genome in the other two lucinid symbiont lineages, Thiohalomonadales and *Thiodiazotropha,* similar to the free-living SOB *A. vinosum*. While the functional implications of these different genome architectures are unknown, the formation or degradation of operon structures profoundly influences gene expression patterns and likely indicates adaptations to diverse lifestyles and environmental conditions (94, 95). Furthermore, although the role of LuxR in regulating sulfur metabolism remains unknown, their widespread association with *sox* genes in the genomes investigated here indicates the potential for interactions between the regulation of sulfur oxidation and population-level metabolic processes and/or host interactions (82).

Interestingly, the only symbiotic SOB whose genome lacks the canonical *soxY* are the symbionts of *Thyasira cf. gouldii*. These encode only the SoxY-S1b homolog, which is also found as a second copy in free-living *Sedimenticola* species as well as in the *Ca*. Sedimenticola endophacoides. The mutation in the “swinging arm” region of the protein compared to the canonical SoxY-S1a suggests potential functional adaptation, possibly related to varying affinities for thiosulfate as proposed for *Sulfurimonas*- and *Sulfurovum*-related Campylobacterota (27–29).

### Potential roles of divergent SoxY homologs in organosulfur, tetrathionate, and alcohol metabolism

**Fig 4:**
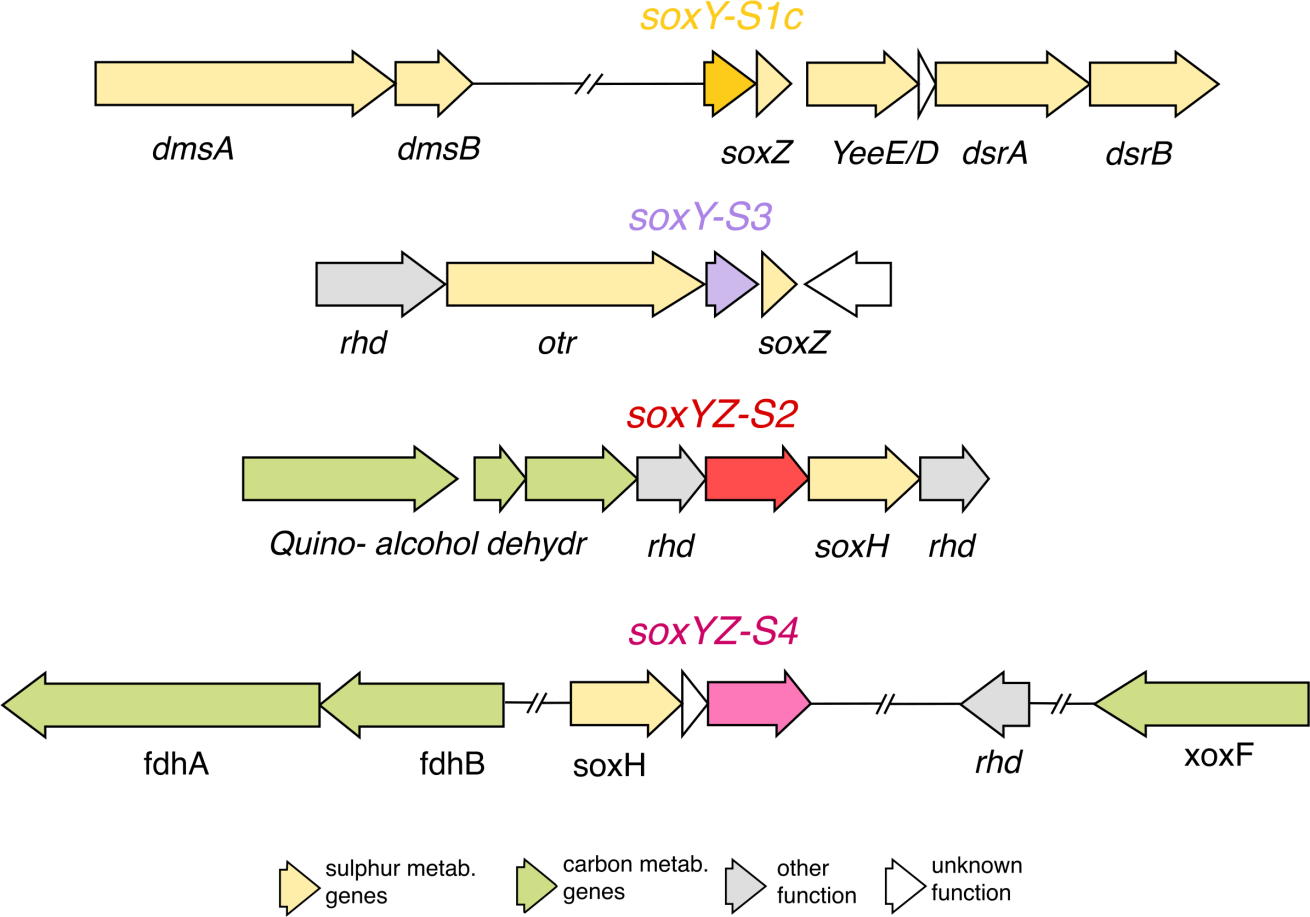
Genetic islands of divergent *soxY* signatures 1b, 1c, 2–4. Yellow coloring represents sulfur metabolism-related genes (including *dmsAB*, dimethyl sulfoxide reductase; YeeE/D, thiosulfate transporter; *dsrAB*, dissimilatory (bi)sulfite reductase, *soxH*); green, carbon related genes (ADH, quinohemoprotein alcohol dehydrogenases; ADH acc., ADH accessory genes; *fdhAB*, formate dehydrogenase; *xoxF*, lanthanide-dependent methanol dehydrogenase); gray, other/multifunctional genes (*rhd*, rhodanese); white genes are of unknown functions.

While the *soxY-S1a* and *soxY-S1b* genes likely play a conserved role in thiosulfate oxidation, the genomic architectures of *soxY-S1c soxYZ-S2, soxY-S3*, and *soxYZ-S4* suggest they may have other functions. The proximity of *soxY-S1c* to genes annotated as DMSO reductases and *soxY-S3* to genes annotated as tetrathionate reductases suggests possible roles in sulfur metabolism distinct from the traditional Sox pathway of thiosulfate oxidation. If so, they possibly do not interact with thiosulfate but may bind to other sulfur compounds (Fig. 4). For example, a divergent SoxY has been proposed to be crucial in transformations of elemental sulfur in *Sulfurimonas* CVO, which is incapable of thiosulphate oxidation (96), and in *Sulfurimonas denitrificans* (30). DMSO could serve as an alternative electron acceptor in low oxygen conditions, while octaheme tetrathionate reductase has been implicated in regulating host-microbe interactions in the anthozoan *Exaiptasia pallida* (97) and the human gut (98). Furthermore, Koch and Dahl (99) reported a distinct link between inorganic (*sox* and *hdr*) and organic sulfur metabolism (*dmsAB* and mtoX) in *Hyphomicrobium denitrificans* (99), which, similarly to lucinid symbionts, encodes for multiple divergent SoxY homologs that have similar sequence signatures.

The fusion genes *soxYZ-S2* and *soxYZ-S4* were in close proximity to *soxH*, a gene potentially associated with sulfur metabolism. In *Sulfurimonas sp*. strain CVO, SoxH was able to compensate for the absence of SoxB by triggering sulfate release from SoxY, thus aiding in elemental sulfur oxidation (96). However, this role for SoxH is improbable for *Thiodiazotropha*, as all metagenome-assembled genomes also encoded a *soxB* gene. Notably, these clades feature SoxYZ fusion proteins, a result of the fusion of both carrier subunit genes, *soxY* and *soxZ,* previously reported in Alphaproteobacteria (71). Sulfur gene fusion was experimentally shown to improve functionality and efficiency, such as *psrB* and *psrC* in *Chlorobium phaeobacteroides* DSM 266, *hdrC* and *hdrB* in green sulfur bacteria, and the TsdB-TsdA fusion protein in *Marichromatium purpuratum* (100, 101). In contrast to *soxY-S1c* and *soxY-S3*, the genes flanking *soxYZ-S2* and *soxYZ-S4* mainly have predicted functions in carbon metabolism; *soxYZ-S2* was associated with a PQQ-dependent quino(hemo)protein alcohol dehydrogenase and *soxYZ-S4* with formate and methanol dehydrogenases. It is noteworthy that clades 2 (SoxYZ-S2) and 4 (SoxYZ-S4) also contained sequences from bacteria that cannot oxidize thiosulfate or only do so in the presence of other substrates. These lines of evidence suggest that SoxYZ-S2 and SoxYZ-S4 are unlikely to be directly involved in sulfur metabolism but could instead play a role in carbon metabolism.

**Fig 5:**
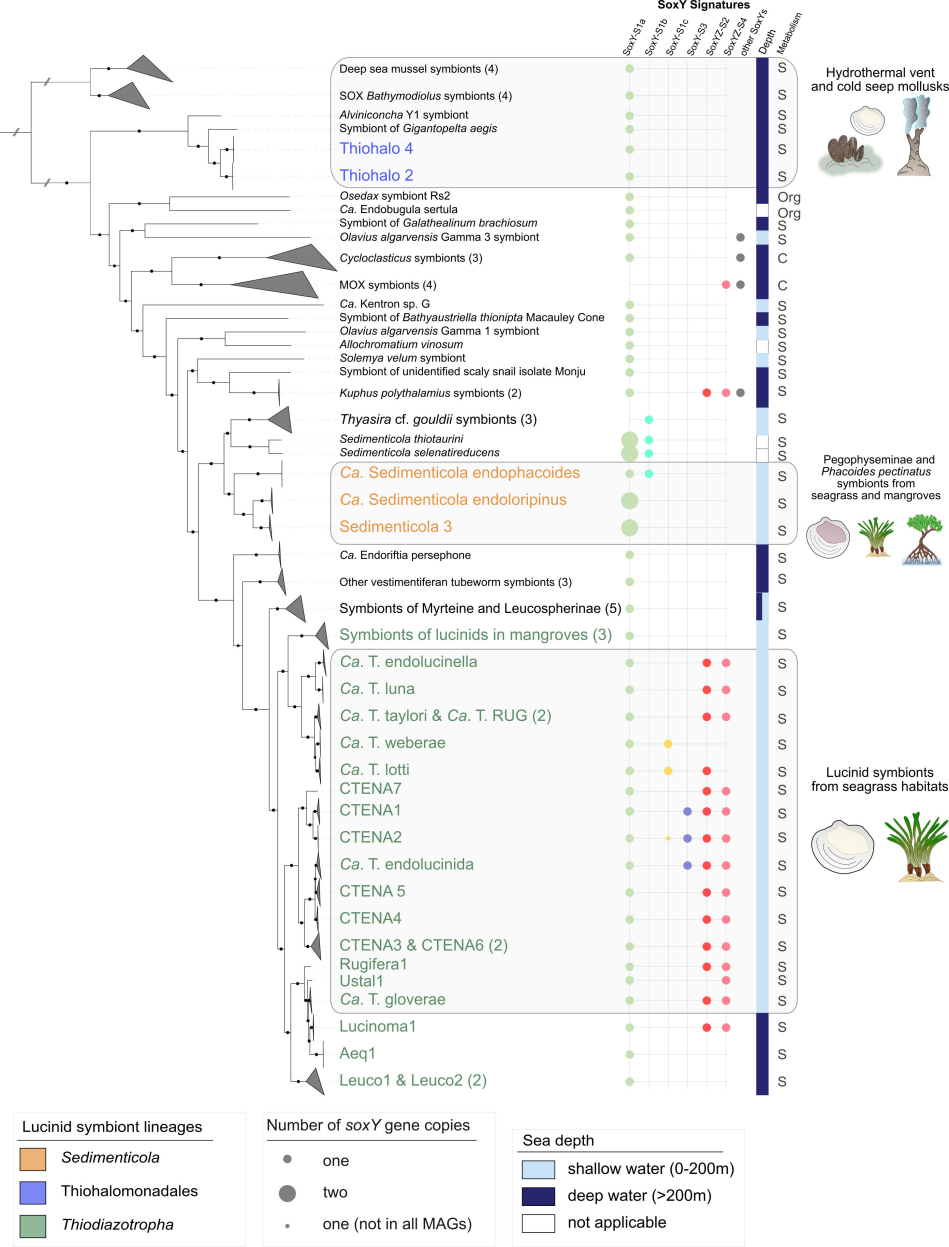
Maximum likelihood phylogenetic tree of symbiotic bacteria reconstructed from GTDB’s multisequence alignment (as in Fig. 1) indicating the host and environmental context. Lucinid symbiont lineages are marked by the following colors: Thiohalomonadales, blue; *Sedimenticola*, yellow; *Thiodiazotropha*, green. The number of symbiont species in each clade is indicated in brackets. The presence and absence of the symbiotic SoxY signatures are marked by colored circles with colors following the legend and previous description. Gray circles show organisms encoding SoxYs with signatures different to those marked in color. The first column from the right indicates the main substrate used for energy metabolism: S, sulfur; Org, complex organic compounds; C, methane and short-chain alkanes.

The discovery that growth on thiosulfate can inhibit methanol degradation, but not formate degradation in *Hyphomicrobium denitrificans* XT, further suggests there are potentially unexplored links between carbon and inorganic sulfur metabolic pathways (78). Interestingly, *H. denitrificans* XT also has a gene encoding for SoxYZ that belonged to clade 4 shared the same swinging-arm sequence as the *Thiodiazotopha* SoxYZ-S4. The synteny of these *soxYZ-S4* and carbon metabolism genes was also conserved in the *Kuphus polythalamia* symbiont and the methanotrophic bathymodiolin symbionts. It is noteworthy that the *soxYZ-S2* and *soxYZ-S4* genes were prevalent in *Thiodiazotropha* symbiont species associated with lucinids from seagrass beds, which are potentially a rich source of alcohols such as methanol [Fig. 5; (102)]. The detoxifying role of SoxYZ-S4 in protecting methanol dehydrogenase from thiosulfate during growth on methanol could be especially beneficial during the free-living phase of *Thiodiazotropha* symbionts in methanol-rich environments like seagrass beds (103). The conservation of genomic links between *soxYZ-S4* and genes for methanol and formate metabolism across multiple bacterial lineages leads us to further speculate that *soxYZ-S4* genes were co-opted to enhance the efficiency of these metabolic pathways.

The co-organization of genes with diverse metabolic functions is a common phenomenon. Operon-driven adaptive evolution often rapidly incorporates new genes, favoring the emergence of single complex promoters over two independent ones. This can lead to operons containing seemingly unrelated genes that are nevertheless co-regulated as they are required under the same environmental conditions (104–106). For instance, conserved operons may include genes for ribosomal proteins and central metabolism, both essential for growth rate regulation (105). The high-sequence divergence of the *soxYZ-S2* and *soxYZ-S4* genes, their fusion with *soxZ*, and their conserved genomic synteny with genes involved in carbon metabolism together indicate that these genes likely originate from horizontal gene transfer (HGT) rather than a recent gene duplication event. This hypothesis is further supported by the presence of genes from diverse bacterial phyla in *soxYZ* clades 2 and 4, the incongruence between the topology of these branches with the phylogenetic relationships of the bacteria, and the presence of *soxYZ* genes in clade 4 that originated from marine viruses, known agents of HGT (Fig. 2; Fig. S4) (49). The versatile ability of SoxY proteins to bind to a range of sulfur compounds (27, 28, 30, 75) provides the foundations that may preadapt these proteins for the evolution of novel functions (107). The co-occurrence of *soxY* genes with various gene families, including genes with established roles in sulfur metabolism within symbiotic SOB, as well as novel candidates like octaheme tetrathionate reductase and genes linked to carbon metabolism (e.g., alcohol, formate, and methanol dehydrogenases), suggests the functions of proteins from this family may be more diverse than previously assumed (75). These new metabolic traits not only support symbionts’ survival in the environment during their free-living stage but are also potentially beneficial during symbiosis. Indeed, adaptations acquired by *Vibrio fischeri* living free in the environment can influence host-microbe interactions and even enhance fitness, while *V. fischeri* is associated with a host (108–110).

#### SoxY diversification shapes ecological strategies and symbiotic adaptations in sulfur-oxidizing bacteria

The lucinid symbioses, characterized by a unique diversity of hosts, habitats, and horizontal symbiont transmission mode, present a unique opportunity for investigating how the interplay between the environment, the host, and symbiont genomic and metabolic adaptations collectively shapes holobiont ecology. Symbiotic SOB, much like their free-living counterparts, have evolved several distinct ecological strategies. They range from “specialists,” which are adapted to grow most efficiently on a restricted range of substrates, and which thrive in highly specific symbiotic relationships with only one or a few host species or unique environments (e.g., hydrothermal vents, mangroves). An example of specialization can be seen in the *Lucinoma aequizonata* symbiosis; this lucinid occurs in oxygen minimum zones and exclusively associates with one *Ca*. Thiodiazotropha species that is uniquely distinguished by its reduced genome size (31). At the other end of the spectrum are “generalists” that can use a wider range of substrates for energy generation, presumably enabling them to colonize a wider range of environments and host species (111). The prime example of the “generalist” strategy would be *Ca*. T. taylori, which has a global distribution and has been detected so far in eight lucinid host species (22). This diversity of ecological strategies likely underpins the high diversity of hosts and habitats in which sulfur-oxidizing symbioses have evolved (Fig. 5) (9, 22).

An interesting pattern emerging from our findings was that symbiotic SOB such as the SUP05 group (also called *Thioglobaceae* [112]) and Thiohalomonadales, which encoded only the canonical SoxY-S1a but not the divergent types, tended to inhabit “extreme” environments such as deep-sea hydrothermal vents, mangroves, or oxygen minimum zones (Fig. 5). On the other hand, SOB symbionts with both canonical and divergent SoxY homologs were associated with lucinids from shallow water seagrass beds or organic-rich sediments with abundant plant debris in the case of the *K. polythalamius* symbionts (Fig. 5). The correlation between specific environments and the diversity of the SoxY repertoire supports the idea of specialization in the symbiotic SOB lacking divergent SoxY proteins (Fig. S9 and S10). In contrast, symbionts encoding several distinct *soxY* genes were only ever found in seagrass beds. Seagrass sediments may be chemically and physically more complex than other environments inhabited by hosts of chemosynthetic symbionts such as “bare” sediments and hydrothermal vents. We hypothesize that expansion of the *soxY* gene family in these symbionts may be one genomic mechanism that underpins their flexibility in associating with a range of hosts and may also underpin the adaptation of the holobiont to complex, fluctuating environments. The proposed function of some divergent SoxYs in C1 or organosulfur metabolism, compounds that are typically enriched in seagrass environments, is consistent with this (102).

The Sedimenticola3 symbiont clade was an interesting exception to these patterns. Despite the operon-like organization of the Sedimenticola3 *sox* genes, a hallmark of specialization on thiosulfate as a substrate, members of this lineage were found in association with lucinids inhabiting diverse shallow-water environments worldwide, such as seagrass, coral reef, and mangrove sediments (Fig. 5). It is noteworthy that the Sedimenticola3 symbionts are almost exclusively restricted to hosts from one lucinid subfamily, Pegophyseminae, where others from the *Thiodiazotropha* genus are typically more flexible and can associate with host species from more than one subfamily. Clams from the family Pegophyseminae also typically have unique morphological features not shared by species from other lucinid subfamilies, which may affect the microniches experienced by their symbionts (86, 113–115). Their specificity for the Pegophyseminae, lack of divergent SoxYs, and operon-like *sox* gene organization lead us to propose that the Sedimenticola3 symbionts specialize primarily on thiosulfate oxidation to obtain energy. If this is the case, it is further interesting to speculate that the Sedimenticola3 and *Ca*. Thiodiazotropha bacteria may occupy distinct niches in the external environment during their free-living phase.

### Conclusions

Our findings suggest *soxY* gene family expansion and diversification, a prominent genomic feature of some symbiotic and free-living SOB, could contribute to metabolic flexibility and a “generalist” ecological strategy for exploiting a larger range of energy sources. We hypothesize that some of these divergent, non-canonical SoxY proteins may carry out novel functions, e.g., in carbon or organosulfur metabolism. Considering that pure cultures are available for some free-living SOB that encode a suite of divergent *soxY* genes, such as *Hyphomicrobium denitrificans* with three *soxY* copies, these hypotheses could be tested experimentally. Unfortunately, most of the genomes available in public databases, and those we generated here, are still not closed. It is therefore challenging to investigate the mechanisms responsible for the expansion of the *soxY* gene family based on the data currently available. However, one intriguing observation was that a SoxY encoded in a viral genome fell into a clade containing lucinid symbiont SoxYs (SoxYZ-S4). Sox genes, including SoxYZ fusion proteins, are commonly found in viral genomes (49). Horizontal acquisition of divergent SoxY(Z) proteins might therefore be one mechanism that could explain their expansion, although we currently have no information about viruses that could infect symbiotic SOB. The lack of congruence between SoxY(Z) proteins and symbiont phylogenies would be consistent with a role of horizontal gene transfer in *soxY* gene family expansion.

## Data Availability

The MAGs recovered and their respective read libraries were deposited in the NCBI BioProject PRJNA1046055. The Biosample accession numbers are SAMN39467937–SAMN39468004. The BioSample accession numbers for the MAGs are SAMN38471244–SAMN38471314, and the accession numbers for the corresponding raw reads are SRR28146002–SRR28145954.

## SUPPLEMENTAL MATERIAL

Supplemental figures msystems.01135-23-s0001.pdf Figures S1 to S10.

Supplemental text msystems.01135-23-s0002.docx Supplemental methods and results.

Table S1 msystems.01135-23-s0003.xlsx Metadata for new MAGs (NCBI submitted MAGs): sample name, host, site, collector.

Table S2 msystems.01135-23-s0004.xlsx Statistics and GTDB classification of all MAGs used in this study.

Table S3 msystems.01135-23-s0005.xlsx Sulfur proteins used in the sulfur metabolism search.

Table S4 msystems.01135-23-s0006.xlsx MAGs used in gene architecture analysis.

Table S5 msystems.01135-23-s0007.xlsx ANI matrix covering of symbiotic MAGs used in this study.

Table S6 msystems.01135-23-s0008.xlsx Presence or absence of sulfur metabolism genes.

ASM does not own the copyrights to Supplemental Material that may be linked to, or accessed through, an article. The authors have granted ASM a non-exclusive, world-wide license to publish the Supplemental Material files. Please contact the corresponding author directly for reuse.

## References

[1] Canfield DE. 1999. The evolution of the sulfur cycle. American J Sci 299:697–723. doi:10.2475/ajs.299.7-9.697

[2] Friedrich CG, Bardischewsky F, Rother D, Quentmeier A, Fischer J. 2005. Prokaryotic sulfur oxidation. Curr Opin Microbiol 8:253–259. doi:10.1016/j.mib.2005.04.005 15939347

[3] Dahl C. 2020. A biochemical view on the biological sulfur cycle, p 55–96. In Environmental technologies to treat sulphur pollution: principles and engineering

[4] Dunker R, Røy H, Kamp A, Jørgensen BB. 2011. Motility patterns of filamentous sulfur bacteria, Beggiatoa spp. FEMS Microbiol Ecol 77:176–185. doi:10.1111/j.1574-6941.2011.01099.x 21446951

[5] Sorokin DY, Kuenen JG, Jetten MS. 2001. Denitrification at extremely high pH values by the alkaliphilic, obligately chemolithoautotrophic, sulfur-oxidizing bacterium Thioalkalivibrio denitrificans strain ALJD. Arch Microbiol 175:94–101. doi:10.1007/s002030000210 11285746

[6] Lenk S, Moraru C, Hahnke S, Arnds J, Richter M, Kube M, Reinhardt R, Brinkhoff T, Harder J, Amann R, Mußmann M. 2012. Roseobacter clade bacteria are abundant in coastal sediments and encode a novel combination of sulfur oxidation genes. ISME J 6:2178–2187. doi:10.1038/ismej.2012.66 22739490 PMC3504970

[7] Childress JJ, Girguis PR. 2011. The metabolic demands of endosymbiotic chemoautotrophic metabolism on host physiological capacities. J Exp Biol 214:312–325. doi:10.1242/jeb.049023 21177951

[8] Dando P, Ridgway S, Spiro B. 1994. Sulphide “mining” by Lucinid bivalve molluscs: demonstrated by stable sulphur isotope measurements and experimental models. Mar Ecol Prog Ser. 107:169–175. doi:10.3354/meps107169

[9] Dubilier N, Bergin C, Lott C. 2008. Symbiotic diversity in marine animals: the art of harnessing chemosynthesis. Nat Rev Microbiol 6:725–740. doi:10.1038/nrmicro1992 18794911

[10] Herry A, Diouris M, Le Pennec M. 1989. Chemoautotrophic symbionts and translocation of fixed carbon from bacteria to host tissues in the littoral bivalve Loripes lucinalis (Lucinidae). Marine Bio 101:305–312. doi:10.1007/BF00428126

[11] Cavanaugh CM, McKiness ZP, Newton ILG, Stewart FJ. 2013. Marine chemosynthetic symbioses, p. 579–607. The prokaryotes: prokaryotic biology and symbiotic associations. Springer Berlin Heidelberg, Berlin, Heidelberg.

[12] Kleiner M, Petersen JM, Dubilier N. 2012. Convergent and divergent evolution of metabolism in sulfur-oxidizing symbionts and the role of horizontal gene transfer. Curr Opin Microbiol 15:621–631. doi:10.1016/j.mib.2012.09.003 23068075

[13] Miller IJ, Weyna TR, Fong SS, Lim-Fong GE, Kwan JC. 2016. Single sample resolution of rare microbial dark matter in a marine invertebrate metagenome. Sci Rep 6:34362. doi:10.1038/srep34362 27681823 PMC5041132

[14] Seah BKB, Antony CP, Huettel B, Zarzycki J, Schada von Borzyskowski L, Erb TJ, Kouris A, Kleiner M, Liebeke M, Dubilier N, Gruber-Vodicka HR. 2019. Sulfur-oxidizing symbionts without canonical genes for autotrophic CO_2_ fixation. mBio 10:e01112-19. doi:10.1128/mBio.01112-19 31239380 PMC6593406

[15] Bright M, Bulgheresi S. 2010. A complex journey: transmission of microbial symbionts. Nat Rev Microbiol 8:218–230. doi:10.1038/nrmicro2262 20157340 PMC2967712

[16] Taylor JD, Glover EA. 2006. Lucinidae (bivalvia)–the most diverse group of chemosymbiotic molluscs. Zool J Linn Soc. 148:421–438. doi:10.1111/j.1096-3642.2006.00261.x

[17] Brissac T, Gros O, Merçot H. 2009. Lack of endosymbiont release by two Lucinidae (Bivalvia) of the genus Codakia: consequences for symbiotic relationships. FEMS Microbiol Ecol 67:261–267. doi:10.1111/j.1574-6941.2008.00626.x 19120467

[18] Frenkiel L, Mouëza M. 1995. Gill ultrastructure and symbiotic bacteria in Codakia orbicularis (Bivalvia, lucinidae). Zoomorphology 115:51–61. doi:10.1007/BF00397934

[19] Petersen JM, Wentrup C, Verna C, Knittel K, Dubilier N. 2012. Origins and evolutionary flexibility of chemosynthetic symbionts from deep-sea animals. Biol Bull 223:123–137. doi:10.1086/BBLv223n1p123 22983038

[20] Harada M, Yoshida T, Kuwahara H, Shimamura S, Takaki Y, Kato C, Miwa T, Miyake H, Maruyama T. 2009. Expression of genes for sulfur oxidation in the intracellular chemoautotrophic symbiont of the deep-sea bivalve Calyptogena okutanii. Extremophiles 13:895–903. doi:10.1007/s00792-009-0277-8 19730970

[21] Stewart FJ, Dmytrenko O, Delong EF, Cavanaugh CM. 2011. Metatranscriptomic analysis of sulfur oxidation genes in the endosymbiont of Solemya velum. Front Microbiol 2:134. doi:10.3389/fmicb.2011.00134 21738524 PMC3125697

[22] Osvatic JT, Wilkins LGE, Leibrecht L, Leray M, Zauner S, Polzin J, Camacho Y, Gros O, van Gils JA, Eisen JA, Petersen JM, Yuen B. 2021. Global biogeography of chemosynthetic symbionts reveals both localized and globally distributed symbiont groups. Proc Natl Acad Sci U S A 118:e2104378118. doi:10.1073/pnas.2104378118 34272286 PMC8307296

[23] Wasmund K, Mußmann M, Loy A. 2017. The life sulfuric: microbial ecology of sulfur cycling in marine sediments. Environ Microbiol Rep 9:323–344. doi:10.1111/1758-2229.12538 28419734 PMC5573963

[24] Kondrashov FA. 2012. Gene duplication as a mechanism of genomic adaptation to a changing environment. Proc Biol Sci 279:5048–5057. doi:10.1098/rspb.2012.1108 22977152 PMC3497230

[25] Friedrich CG, Quentmeier A, Bardischewsky F, Rother D, Kraft R, Kostka S, Prinz H. 2000. Novel genes coding for lithotrophic sulfur oxidation of Paracoccus pantotrophus GB17. J Bacteriol 182:4677–4687. doi:10.1128/JB.182.17.4677-4687.2000 10940005 PMC111341

[26] Quentmeier A, Friedrich CG. 2001. The cysteine residue of the SoxY protein as the active site of protein-bound sulfur oxidation of Paracoccus pantotrophus GB17. FEBS Lett. 503:168–172. doi:10.1016/s0014-5793(01)02727-2 11513876

[27] Meier DV, Pjevac P, Bach W, Hourdez S, Girguis PR, Vidoudez C, Amann R, Meyerdierks A. 2017. Niche partitioning of diverse sulfur-oxidizing bacteria at hydrothermal vents. ISME J 11:1545–1558. doi:10.1038/ismej.2017.37 28375213 PMC5520155

[28] Pjevac P, Meier DV, Markert S, Hentschker C, Schweder T, Becher D, Gruber-Vodicka HR, Richter M, Bach W, Amann R, Meyerdierks A. 2018. Metaproteogenomic profiling of microbial communities colonizing actively venting hydrothermal chimneys. Front Microbiol 9:680. doi:10.3389/fmicb.2018.00680 29696004 PMC5904459

[29] Meier D. 2016. Bacterial niche adaptation at hydrothermal vents. Universität Bremen.

[30] Götz F, Pjevac P, Markert S, McNichol J, Becher D, Schweder T, Mussmann M, Sievert SM. 2019. Transcriptomic and proteomic insight into the mechanism of cyclooctasulfur- versus thiosulfate-oxidation by the chemolithoautotroph Sulfurimonas denitrificans. Environ Microbiol 21:244–258. doi:10.1111/1462-2920.14452 30362214

[31] Osvatic JT, Yuen B, Kunert M, Wilkins L, Hausmann B, Girguis P, Lundin K, Taylor J, Jospin G, Petersen JM. 2023. Gene loss and symbiont switching during adaptation to the deep sea in a globally distributed symbiosis. ISME J 17:453–466. doi:10.1038/s41396-022-01355-z 36639537 PMC9938160

[32] Bushnell B. 2014. BBMap: a fast, accurate, splice-aware aligner. Lawrence Berkeley National Lab.(LBNL), Berkeley, CA (United States).

[33] Nurk S, Meleshko D, Korobeynikov A, Pevzner PA. 2017. metaSPAdes: a new versatile metagenomic assembler. Genome Res. 27:824–834. doi:10.1101/gr.213959.116 28298430 PMC5411777

[34] Nurk S, Bankevich A, Antipov D, Gurevich A, Korobeynikov A, Lapidus A, Prjibelsky A, Pyshkin A, Sirotkin A, Sirotkin Y. 2013. “Assembling Genomes and mini-Metagenomes from highly Chimeric reads” Research in Computational Molecular Biology: 17th Annual International Conference, RECOMB, p 158–170 Springer, Beijing, China. doi:10.1007/978-3-642-37195-0 PMC3791033 24093227

[35] Li H, Handsaker B, Wysoker A, Fennell T, Ruan J, Homer N, Marth G, Abecasis G, Durbin R, 1000 Genome Project Data Processing Subgroup. 2009. The sequence alignment/map format and SAMtools. Bioinformatics 25:2078–2079. doi:10.1093/bioinformatics/btp352 19505943 PMC2723002

[36] Eren AM, Esen ÖC, Quince C, Vineis JH, Morrison HG, Sogin ML, Delmont TO. 2015. Anvi’O: an advanced analysis and visualization platform for ‘omics data. PeerJ 3:e1319. doi:10.7717/peerj.1319 26500826 PMC4614810

[37] Alneberg J, Bjarnason BS, de Bruijn I, Schirmer M, Quick J, Ijaz UZ, Lahti L, Loman NJ, Andersson AF, Quince C. 2014. Binning metagenomic contigs by coverage and composition. Nat Methods 11:1144–1146. doi:10.1038/nmeth.3103 25218180

[38] Kang DD, Li F, Kirton E, Thomas A, Egan R, An H, Wang Z. 2019. MetaBAT 2: an adaptive binning algorithm for robust and efficient genome reconstruction from metagenome assemblies. PeerJ 7:e7359. doi:10.7717/peerj.7359 31388474 PMC6662567

[39] Olm MR, Brown CT, Brooks B, Banfield JF. 2017. dRep: a tool for fast and accurate genomic comparisons that enables improved genome recovery from metagenomes through de-replication. ISME J 11:2864–2868. doi:10.1038/ismej.2017.126 28742071 PMC5702732

[40] Parks DH, Imelfort M, Skennerton CT, Hugenholtz P, Tyson GW. 2015. CheckM: assessing the quality of microbial genomes recovered from isolates, single cells, and metagenomes. Genome Res 25:1043–1055. doi:10.1101/gr.186072.114 25977477 PMC4484387

[41] Weissgerber T, Zigann R, Bruce D, Chang Y-J, Detter JC, Han C, Hauser L, Jeffries CD, Land M, Munk AC, Tapia R, Dahl C. 2011. Complete genome sequence of Allochromatium vinosum DSM 180T. Stand Genomic Sci 5:311–330. doi:10.4056/sigs.2335270 22675582 PMC3368242

[42] Flood BE, Jones DS, Bailey JV. 2015. Complete genome sequence of Sedimenticola thiotaurini strain SIP-G1, a polyphosphate- and polyhydroxyalkanoate-accumulating sulfur-oxidizing gammaproteobacterium isolated from salt marsh sediments. Genome Announc 3:00671–15. doi:10.1128/genomeA.00671-15 PMC4472907 26089430

[43] Narasingarao P, Häggblom MM. 2006. Sedimenticola selenatireducens, gen. nov., sp. nov., an anaerobic selenate-respiring bacterium isolated from estuarine sediment. Syst Appl Microbiol 29:382–388. doi:10.1016/j.syapm.2005.12.011 16427757

[44] Tanabe TS, Dahl C. 2023. HMSS2: an advanced tool for the analysis of sulphur metabolism, including organosulphur compound transformation, in genome and metagenome assemblies. Mol Ecol Resour 23:1930–1945. doi:10.1111/1755-0998.13848 37515475

[45] Mahram A, Herbordt MC. 2015. NCBI BLASTP on high-performance reconfigurable computing systems. ACM Trans Reconfigurable Technol Syst 7:1–20. doi:10.1145/2629691 PMC3109751 21660208

[46] Hyatt D, Chen G-L, Locascio PF, Land ML, Larimer FW, Hauser LJ. 2010. Prodigal: prokaryotic gene recognition and translation initiation site identification. BMC Bioinformatics 11:119. doi:10.1186/1471-2105-11-119 20211023 PMC2848648

[47] Huerta-Cepas J, Szklarczyk D, Heller D, Hernández-Plaza A, Forslund SK, Cook H, Mende DR, Letunic I, Rattei T, Jensen LJ, von Mering C, Bork P. 2019. EggNOG 5.0: a hierarchical, functionally and phylogenetically annotated orthology resource based on 5090 organisms and 2502 viruses. Nucleic Acids Res. 47:D309–D314. doi:10.1093/nar/gky1085 30418610 PMC6324079

[48] Finn RD, Clements J, Eddy SR. 2011. HMMER web server: interactive sequence similarity searching. Nucleic Acids Res. 39:W29–37. doi:10.1093/nar/gkr367 21593126 PMC3125773

[49] Kieft K, Zhou Z, Anderson RE, Buchan A, Campbell BJ, Hallam SJ, Hess M, Sullivan MB, Walsh DA, Roux S, Anantharaman K. 2021. Ecology of inorganic sulfur auxiliary metabolism in widespread bacteriophages. Nat Commun 12:3503. doi:10.1038/s41467-021-23698-5 34108477 PMC8190135

[50] Jones P, Binns D, Chang HY, Fraser M, Li W, McAnulla C, McWilliam H, Maslen J, Mitchell A, Nuka G, Pesseat S, Quinn AF, Sangrador-Vegas A, Scheremetjew M, Yong SY, Lopez R, Hunter S. 2014. InterProScan 5: genome-scale protein function classification. Bioinformatics 30:1236–1240. doi:10.1093/bioinformatics/btu031 24451626 PMC3998142

[51] Shen W, Le S, Li Y, Hu F. 2016. Seqkit: a cross-platform and ultrafast toolkit for FASTA/Q file manipulation. PLoS One 11:e0163962. doi:10.1371/journal.pone.0163962 27706213 PMC5051824

[52] Kumar S, Stecher G, Li M, Knyaz C, Tamura K. 2018. MEGA X: molecular evolutionary genetics analysis across computing platforms. Mol Biol Evol 35:1547–1549. doi:10.1093/molbev/msy096 29722887 PMC5967553

[53] Nguyen L-T, Schmidt HA, von Haeseler A, Minh BQ. 2015. IQ-TREE: a fast and effective stochastic algorithm for estimating maximum-likelihood phylogenies. Mol Biol Evol 32:268–274. doi:10.1093/molbev/msu300 25371430 PMC4271533

[54] Minh BQ, Schmidt HA, Chernomor O, Schrempf D, Woodhams MD, von Haeseler A, Lanfear R. 2020. IQ-TREE 2: new models and efficient methods for phylogenetic inference in the genomic era. Mol Biol Evol 37:1530–1534. doi:10.1093/molbev/msaa015 32011700 PMC7182206

[55] Kalyaanamoorthy S, Minh BQ, Wong TKF, von Haeseler A, Jermiin LS. 2017. ModelFinder: fast model selection for accurate phylogenetic estimates. Nat Methods 14:587–589. doi:10.1038/nmeth.4285 28481363 PMC5453245

[56] Hoang DT, Chernomor O, von Haeseler A, Minh BQ, Vinh LS. 2018. UFBoot2: improving the ultrafast bootstrap approximation. Mol Biol Evol 35:518–522. doi:10.1093/molbev/msx281 29077904 PMC5850222

[57] Letunic I, Bork P. 2021. Interactive tree of life (iTOL) V5: An online tool for Phylogenetic tree display and annotation. Nucleic Acids Res. 49:W293–W296. doi:10.1093/nar/gkab301 33885785 PMC8265157

[58] Matsen FA, Kodner RB, Armbrust EV. 2010. Pplacer: linear time maximum-likelihood and bayesian phylogenetic placement of sequences onto a fixed reference tree. BMC Bioinformatics 11:538. doi:10.1186/1471-2105-11-538 21034504 PMC3098090

[59] Jain C, Rodriguez-R LM, Phillippy AM, Konstantinidis KT, Aluru S. 2018. High throughput ANI analysis of 90K prokaryotic genomes reveals clear species boundaries. Nat Commun 9:5114. doi:10.1038/s41467-018-07641-9 30504855 PMC6269478

[60] Price MN, Dehal PS, Arkin AP. 2010. FastTree 2 – approximately maximum-likelihood trees for large alignments. PLoS One 5:e9490. doi:10.1371/journal.pone.0009490 20224823 PMC2835736

[61] Eddy SR. 2011. Accelerated profile HMM searches. PLoS Comput Biol 7:e1002195. doi:10.1371/journal.pcbi.1002195 22039361 PMC3197634

[62] Ondov BD, Treangen TJ, Melsted P, Mallonee AB, Bergman NH, Koren S, Phillippy AM. 2016. Mash: fast genome and metagenome distance estimation using MinHash. Genome Biol. 17:132. doi:10.1186/s13059-016-0997-x 27323842 PMC4915045

[63] Aziz RK, Bartels D, Best AA, DeJongh M, Disz T, Edwards RA, Formsma K, Gerdes S, Glass EM, Kubal M, et al.. 2008. The RAST server: rapid annotations using subsystems technology. BMC Genomics 9:75. doi:10.1186/1471-2164-9-75 18261238 PMC2265698

[64] Brettin T, Davis JJ, Disz T, Edwards RA, Gerdes S, Olsen GJ, Olson R, Overbeek R, Parrello B, Pusch GD, Shukla M, Thomason JA, Stevens R, Vonstein V, Wattam AR, Xia F. 2015. RASTtk: a modular and extensible implementation of the RAST algorithm for building custom annotation pipelines and annotating batches of genomes. Sci Rep 5:8365. doi:10.1038/srep08365 25666585 PMC4322359

[65] Overbeek R, Olson R, Pusch GD, Olsen GJ, Davis JJ, Disz T, Edwards RA, Gerdes S, Parrello B, Shukla M, Vonstein V, Wattam AR, Xia F, Stevens R. 2014. The SEED and the rapid annotation of microbial genomes using subsystems technology (RAST). Nucleic Acids Res. 42:D206–14. doi:10.1093/nar/gkt1226 24293654 PMC3965101

[66] Gish W, States DJ. 1993. Identification of protein coding regions by database similarity search. Nat Genet 3:266–272. doi:10.1038/ng0393-266 8485583

[67] Camacho C, Coulouris G, Avagyan V, Ma N, Papadopoulos J, Bealer K, Madden TL. 2009. BLAST+: architecture and applications. BMC Bioinformatics 10:421. doi:10.1186/1471-2105-10-421 20003500 PMC2803857

[68] Finn RD, Bateman A, Clements J, Coggill P, Eberhardt RY, Eddy SR, Heger A, Hetherington K, Holm L, Mistry J, Sonnhammer ELL, Tate J, Punta M. 2014. Pfam: the protein families database. Nucl Acids Res 42:D222–D230. doi:10.1093/nar/gkt1223 24288371 PMC3965110

[69] Bowers RM, Kyrpides NC, Stepanauskas R, Harmon-Smith M, Doud D, Reddy TBK, Schulz F, Jarett J, Rivers AR, Eloe-Fadrosh EA, et al.. 2017. Minimum information about a single amplified genome (MISAG) and a metagenome-assembled genome (MIMAG) of bacteria and archaea. Nat Biotechnol 35:725–731. doi:10.1038/nbt.3893 28787424 PMC6436528

[70] Dahl C. 2008. Edited by C. G. Friedrich. Microbial sulfur metabolism. Springer, Berlin, Germany.

[71] Ghosh W, Mallick S, DasGupta SK. 2009. Origin of the sox multienzyme complex system in ancient thermophilic bacteria and coevolution of its constituent proteins. Res Microbiol 160:409–420. doi:10.1016/j.resmic.2009.07.003 19616092

[72] Vetter RD. 1985. Elemental sulfur in the gills of three species of clams containing chemoautotrophic symbiotic bacteria: a possible inorganic energy storage compound. Mar Biol. 88:33–42. doi:10.1007/BF00393041

[73] Dahl C, Prange A. 2008. Bacterial sulfur globules: occurrence, structure and metabolism, p 21–51. In Inclusions in prokaryotes. Springer-Verlag, Berlin/Heidelberg.

[74] Hensen D, Sperling D, Trüper HG, Brune DC, Dahl C. 2006. Thiosulphate oxidation in the phototrophic sulphur bacterium Allochromatium vinosum. Mol Microbiol 62:794–810. doi:10.1111/j.1365-2958.2006.05408.x 16995898

[75] Sauvé V, Bruno S, Berks BC, Hemmings AM. 2007. The SoxYZ complex carries sulfur cycle intermediates on a peptide swinging arm. J Biol Chem 282:23194–23204. doi:10.1074/jbc.M701602200 17522046

[76] Rubin-Blum M, Antony CP, Borowski C, Sayavedra L, Pape T, Sahling H, Bohrmann G, Kleiner M, Redmond MC, Valentine DL, Dubilier N. 2017. Short-chain alkanes fuel mussel and sponge Cycloclasticus symbionts from deep-sea gas and oil seeps. Nat Microbiol 2:17093. doi:10.1038/nmicrobiol.2017.93 28628098 PMC5490736

[77] Zhang R-C, Xu X-J, Chen C, Xing D-F, Shao B, Liu W-Z, Wang A-J, Lee D-J, Ren N-Q. 2018. Interactions of functional bacteria and their contributions to the performance in integrated autotrophic and heterotrophic denitrification. Water Res. 143:355–366. doi:10.1016/j.watres.2018.06.053 29986245

[78] Li J, Koch J, Flegler W, Garcia Ruiz L, Hager N, Ballas A, Tanabe TS, Dahl C. 2023. A metabolic puzzle: consumption of C1 compounds and thiosulfate in Hyphomicrobium denitrificans XT. Biochimica et Biophysica Acta (BBA) - Bioenergetics 1864:148932. doi:10.1016/j.bbabio.2022.148932 36367491

[79] Kelly DP, Anthony C, Murrell JC. 2005. Insights into the obligate methanotroph Methylococcus capsulatus. Trends Microbiol. 13:195–198. doi:10.1016/j.tim.2005.03.003 15866035

[80] Fetherston R. 2018. Functional genomics of methylated sulfur compound metabolism in Hyphomicrobium species. University of Warwick.

[81] Gwak J-H, Awala SI, Nguyen N-L, Yu W-J, Yang H-Y, von Bergen M, Jehmlich N, Kits KD, Loy A, Dunfield PeterF, Dahl C, Hyun J-H, Rhee S-K. 2022. Sulfur and methane oxidation by a single microorganism. Proc Natl Acad Sci USA 119. doi:10.1073/pnas.2114799119 PMC9371685 35914169

[82] Patankar AV, González JE. 2009. Orphan LuxR regulators of quorum sensing. FEMS Microbiol Rev 33:739–756. doi:10.1111/j.1574-6976.2009.00163.x 19222586

[83] Sarmiento-Pavía PD, Sosa-Torres ME. 2021. Bioinorganic insights of the PQQ-dependent alcohol dehydrogenases. J Biol Inorg Chem 26:177–203. doi:10.1007/s00775-021-01852-0 33606117

[84] Mowat CG, Rothery E, Miles CS, McIver L, Doherty MK, Drewette K, Taylor P, Walkinshaw MD, Chapman SK, Reid GA. 2004. Octaheme tetrathionate reductase is a respiratory enzyme with novel heme ligation. Nat Struct Mol Biol 11:1023–1024. doi:10.1038/nsmb827 15361860

[85] Atkinson SJ, Mowat CG, Reid GA, Chapman SK. 2007. An octaheme c -type cytochrome from Shewanella oneidensis can reduce nitrite and hydroxylamine. FEBS Lett. 581:3805–3808. doi:10.1016/j.febslet.2007.07.005 17659281

[86] Anderson AE. 1995. Metabolic responses to sulfur in lucinid bivalves. Am Zool 35:121–131. doi:10.1093/icb/35.2.121

[87] Belkin S, Nelson DC, Jannasch HW. 1986. Symbiotic assimilation of Co_2_ in two hydrothermal vent animals. Biol Bull 170:120–121. doi:10.2307/1541384

[88] Andersson DI, Hughes D. 2009. Gene amplification and adaptive evolution in bacteria. Annu Rev Genet 43:167–195. doi:10.1146/annurev-genet-102108-134805 19686082

[89] Sandegren L, Andersson DI. 2009. Bacterial gene amplification: implications for the evolution of antibiotic resistance. Nat Rev Microbiol 7:578–588. doi:10.1038/nrmicro2174 19609259

[90] Elliott KT, Cuff LE, Neidle EL. 2013. Copy number change: evolving views on gene amplification. Future Microbiol 8:887–899. doi:10.2217/fmb.13.53 23841635

[91] Masuda S, Hennecke H, Fischer HM. 2017. Requirements for efficient thiosulfate oxidation in Bradyrhizobium diazoefficiens. Genes (Basel) 8:390. doi:10.3390/genes8120390 29244759 PMC5748708

[92] Friedrich CG, Rother D, Bardischewsky F, Quentmeier A, Fischer J. 2001. Oxidation of reduced inorganic sulfur compounds by bacteria: emergence of a common mechanism?. Appl Environ Microbiol 67:2873–2882. doi:10.1128/AEM.67.7.2873-2882.2001 11425697 PMC92956

[93] Yin H, Zhang X, Li X, He Z, Liang Y, Guo X, Hu Q, Xiao Y, Cong J, Ma L, Niu J, Liu X. 2014. Whole-genome sequencing reveals novel insights into sulfur oxidation in the extremophile Acidithiobacillus thiooxidans. BMC Microbiol 14:179. doi:10.1186/1471-2180-14-179 24993543 PMC4109375

[94] Dandekar T, Snel B, Huynen M, Bork P. 1998. Conservation of gene order: a fingerprint of proteins that physically interact. Trends Biochem Sci 23:324–328. doi:10.1016/s0968-0004(98)01274-2 9787636

[95] Price MN, Arkin AP, Alm EJ. 2006. The life-cycle of operons. PLoS Genet 2:e96. doi:10.1371/journal.pgen.0020096 16789824 PMC1480536

[96] Lahme S, Callbeck CM, Eland LE, Wipat A, Enning D, Head IM, Hubert CRJ. 2020. Comparison of sulfide-oxidizing Sulfurimonas strains reveals a new mode of thiosulfate formation in subsurface environments. Environ Microbiol 22:1784–1800. doi:10.1111/1462-2920.14894 31840396

[97] Krediet CJ, Meyer JL, Gimbrone N, Yanong R, Berzins I, Alagely A, Castro H, Ritchie KB, Paul VJ, Teplitski M. 2014. Interactions between the tropical sea anemone Aiptasia pallida and Serratia marcescens, an opportunistic pathogen of corals. Environ Microbiol Rep 6:287–292. doi:10.1111/1758-2229.12151 24983533

[98] Winter SE, Thiennimitr P, Winter MG, Butler BP, Huseby DL, Crawford RW, Russell JM, Bevins CL, Adams LG, Tsolis RM, Roth JR, Bäumler AJ. 2010. Gut inflammation provides a respiratory electron acceptor for Salmonella. Nature 467:426–429. doi:10.1038/nature09415 20864996 PMC2946174

[99] Koch T, Dahl C. 2018. A novel bacterial sulfur oxidation pathway provides a new link between the cycles of organic and inorganic sulfur compounds. ISME J 12:2479–2491. doi:10.1038/s41396-018-0209-7 29930335 PMC6155103

[100] Frigaard N-U, Dahl C. 2008. Sulfur metabolism in Phototrophic sulfur bacteria, p 103–200. In Advances in microbial physiology 10.1016/S0065-2911(08)00002-7 18929068

[101] Kurth JM, Brito JA, Reuter J, Flegler A, Koch T, Franke T, Klein EM, Rowe SF, Butt JN, Denkmann K, Pereira IAC, Archer M, Dahl C. 2016. Electron accepting units of the diheme cytochrome c TsdA, a bifunctional thiosulfate dehydrogenase/tetrathionate reductase. J Biol Chem 291:24804–24818. doi:10.1074/jbc.M116.753863 27694441 PMC5122753

[102] Adamczyk EM, O’Connor MI, Parfrey LW. 2022. Seagrass (Zostera marina) transplant experiment reveals core microbiota and resistance to environmental change. Mol Ecol 31:5107–5123. doi:10.1111/mec.16641 35933734

[103] Martin BC, Middleton JA, Fraser MW, Marshall IPG, Scholz VV, Hausl B, Schmidt H. 2020. Cutting out the middle clam: lucinid endosymbiotic bacteria are also associated with seagrass roots worldwide. ISME J 14:2901–2905. doi:10.1038/s41396-020-00771-3 32929207 PMC7784995

[104] de Daruvar A, Collado-Vides J, Valencia A. 2002. Analysis of the cellular functions of Escherichia coli operons and their conservation in Bacillus subtilis. J Mol Evol 55:211–221. doi:10.1007/s00239-002-2317-1 12107597

[105] Rogozin IB, Makarova KS, Murvai J, Czabarka E, Wolf YI, Tatusov RL, Szekely LA, Koonin EV. 2002. Connected gene neighborhoods in prokaryotic genomes. Nucleic Acids Res 30:2212–2223. doi:10.1093/nar/30.10.2212 12000841 PMC115289

[106] Price MN, Huang KH, Arkin AP, Alm EJ. 2005. Operon formation is driven by co-regulation and not by horizontal gene transfer. Genome Res 15:809–819. doi:10.1101/gr.3368805 15930492 PMC1142471

[107] Francino MP. 2012. The ecology of bacterial genes and the survival of the new. Int J Evol Biol 2012:394026. doi:10.1155/2012/394026 22900231 PMC3415099

[108] Svensson E, Calsbeek R. 2012. The adaptive landscape in evolutionary biology. Oxford University Press.

[109] Cohen ML, Mashanova EV, Rosen NM, Soto W. 2019. Adaptation to temperature stress by Vibrio fischeri facilitates this microbe’s symbiosis with the Hawaiian bobtail squid (Euprymna scolopes). Evolution 73:1885–1897. doi:10.1111/evo.13819 31397886

[110] Soto W, Travisano M, Tolleson AR, Nishiguchi MK. 2019. Symbiont evolution during the free-living phase can improve host colonization. Microbiology (Reading) 165:174–187. doi:10.1099/mic.0.000756 30648935 PMC7003651

[111] Devictor V, Julliard R, Jiguet F. 2008. Distribution of specialist and generalist species along spatial gradients of habitat disturbance and fragmentation. Oikos 117:507–514. doi:10.1111/j.0030-1299.2008.16215.x

[112] Morris RM, Spietz RL. 2022. The physiology and biogeochemistry of SUP05. Ann Rev Mar Sci 14:261–275. doi:10.1146/annurev-marine-010419-010814 34416125

[113] Kraus DW. 1995. Heme-proteins in sulfide-oxidizing bacteria mollusk symbioses. Am Zool 35:112–120. doi:10.1093/icb/35.2.112

[114] Taylor JD, Glover EA. 2000. Functional anatomy, chemosymbiosis and evolution of the lucinidae. SP 177:207–225. doi:10.1144/GSL.SP.2000.177.01.12

[115] Taylor JD, Glover E. 2021. Biology, evolution and generic review of the chemosymbiotic bivalve family lucinidae. Ray Society, London.

