# Supplementary material for: SoxY gene family expansion underpins adaptation to diverse hosts and environments in symbiotic sulfide oxidizers
Source: mSystems. 2024 May 15;9(6):e01135-23. doi: 10.1128/msystems.01135-23 (PMC11237559; doi:10.1128/msystems.01135-23)
Supplement: Supplemental figures — Figures S1 to S10. [file msystems.01135-23-s0001.pdf]

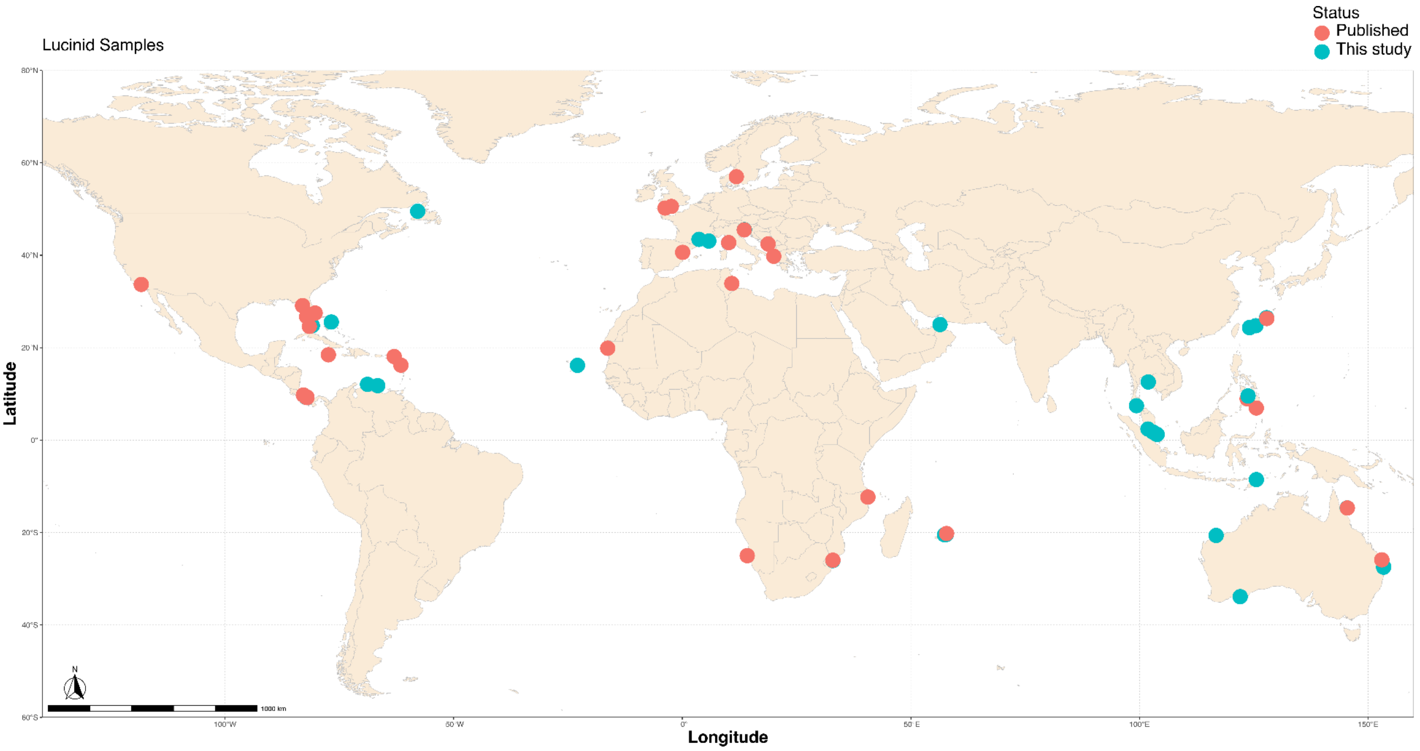

Figure S1: Sampling locations of the samples used in this study. Samples sequenced in this study are labeled in cyan, published genomes are labeled red.

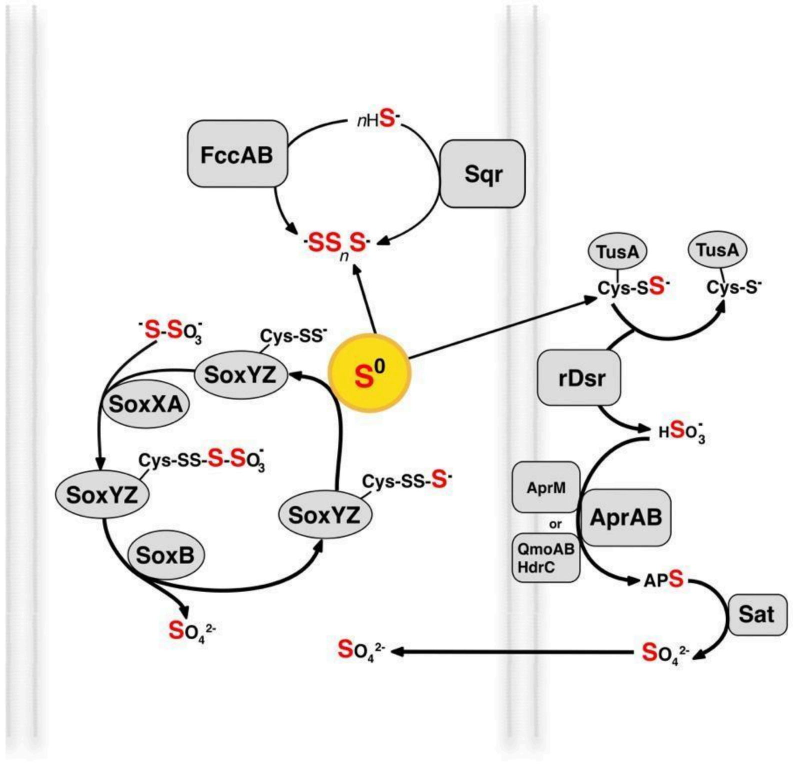

Figure S2: Model of metabolic pathways involved in sulfur oxidation metabolism in symbiotic SOB. All symbiotic SOB encode sulfur oxidation metabolic pathways either identical or of complementary function The only genes that differed encoded the functionally complementary enzymes AprM and QmoAB/HdrBC. S0 sulfur globules, FccAB flavocytochrome c, Sqr sulfide:quinone oxidoreductase, Sox periplasmic thiosulfate oxidizing multienzyme complex, Apr adenosine-5’-phosphosulfate reductase, HdrBC Heterodisulfide reductase, QmoAB quinone-interacting membrane-bound oxidoreductase, Sat dissimilatory ATP sulfurylase.

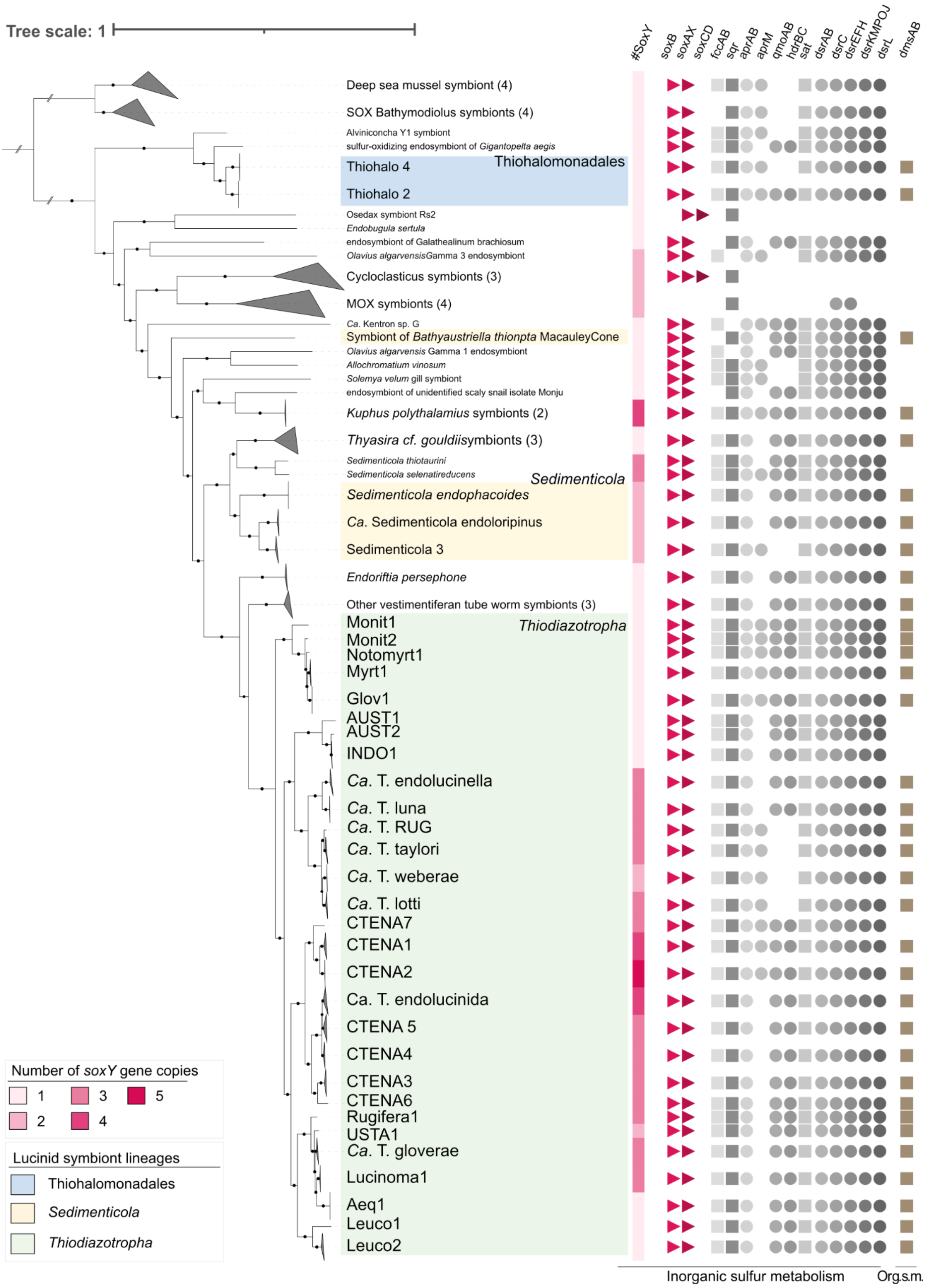

Figure S3: Maximum likelihood phylogenetic tree of symbiotic bacteria reconstructed from GTDB's multisequence alignment using the best-fit model LG+I+G4 and 1000 bootstraps Black circles describe bootstrap support values above 85%. Clades containing symbiont MAGs generated in this study are indicated by asterisks. Number of symbiont species in each of the clades is indicated by the number in brackets. Lucinid symbiont genera are indicated in the following colors: Thiohalomonadales - blue, *Sedimenticola* - yellow, *Thiodiazotropha* - green. The first column describes the number of encoded *soxY*

genes in each symbiont MAG. Further columns describe presence or absence of major sulfur-oxidizing genes within inorganic and organic (Org. s. m.) sulfur metabolism.

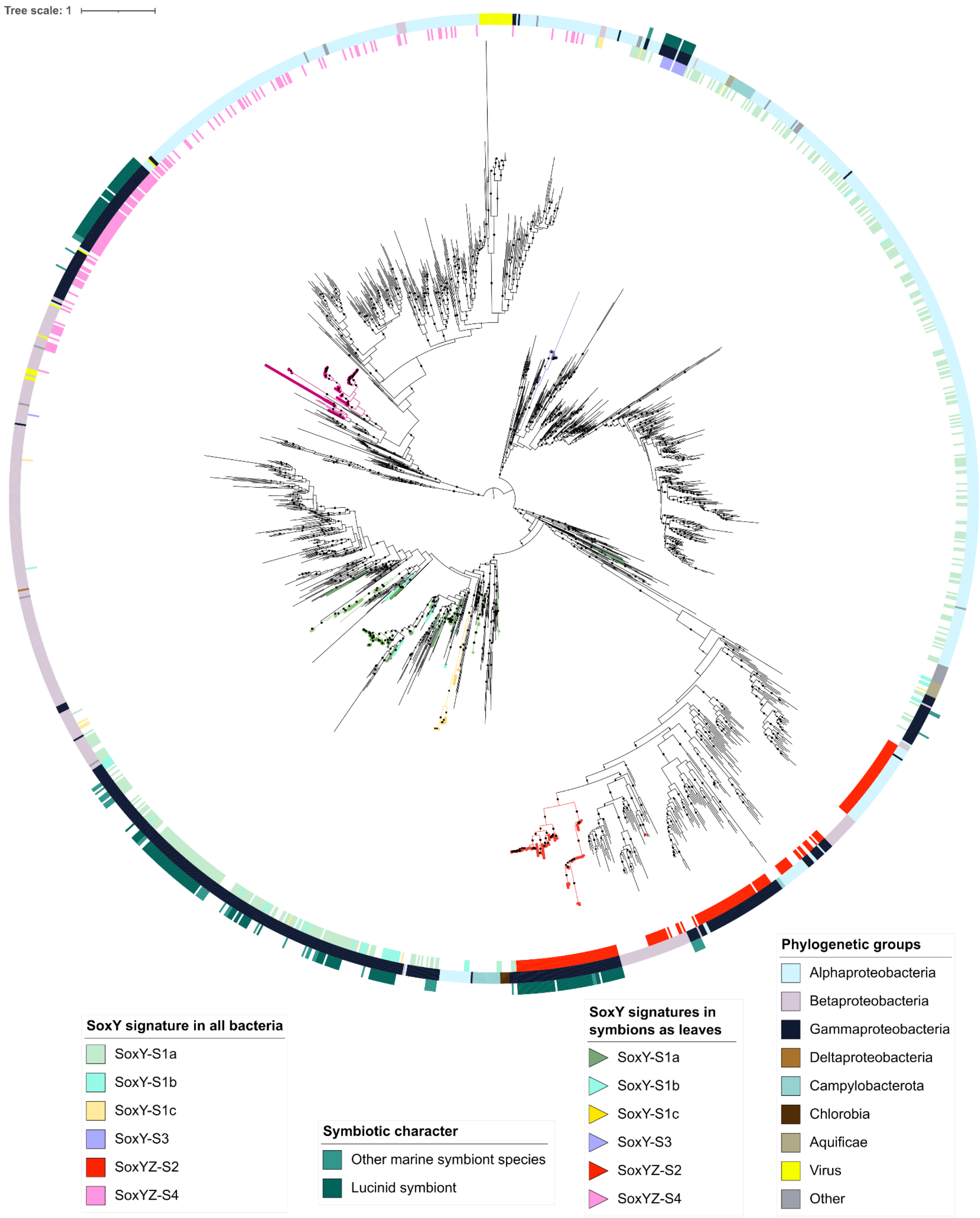

Figure S4: Maximum likelihood tree of SoxY proteins (1631 sequences total) from symbionts and free-living bacteria including sequences from NCBI and Pfam (RP55). Leaves containing symbiotic SoxY have been colored accordingly to the legend. The SoxY sequence signature groups were distinguished by mutations within the sulfur-binding “swinging arm” of the

protein and labelled for free-living and symbiotic bacteria in the inner circle according to the legend. The canonical SoxY sequence with its conserved sulfur-binding cysteine-110 (cys110) is labeled as SoxY-S1a. All bacteria have been classified to the major phylogenetic groups seen in middle circle, according to the legend. Symbiotic sequences have been labelled in the outer circle with dark and light cyan.

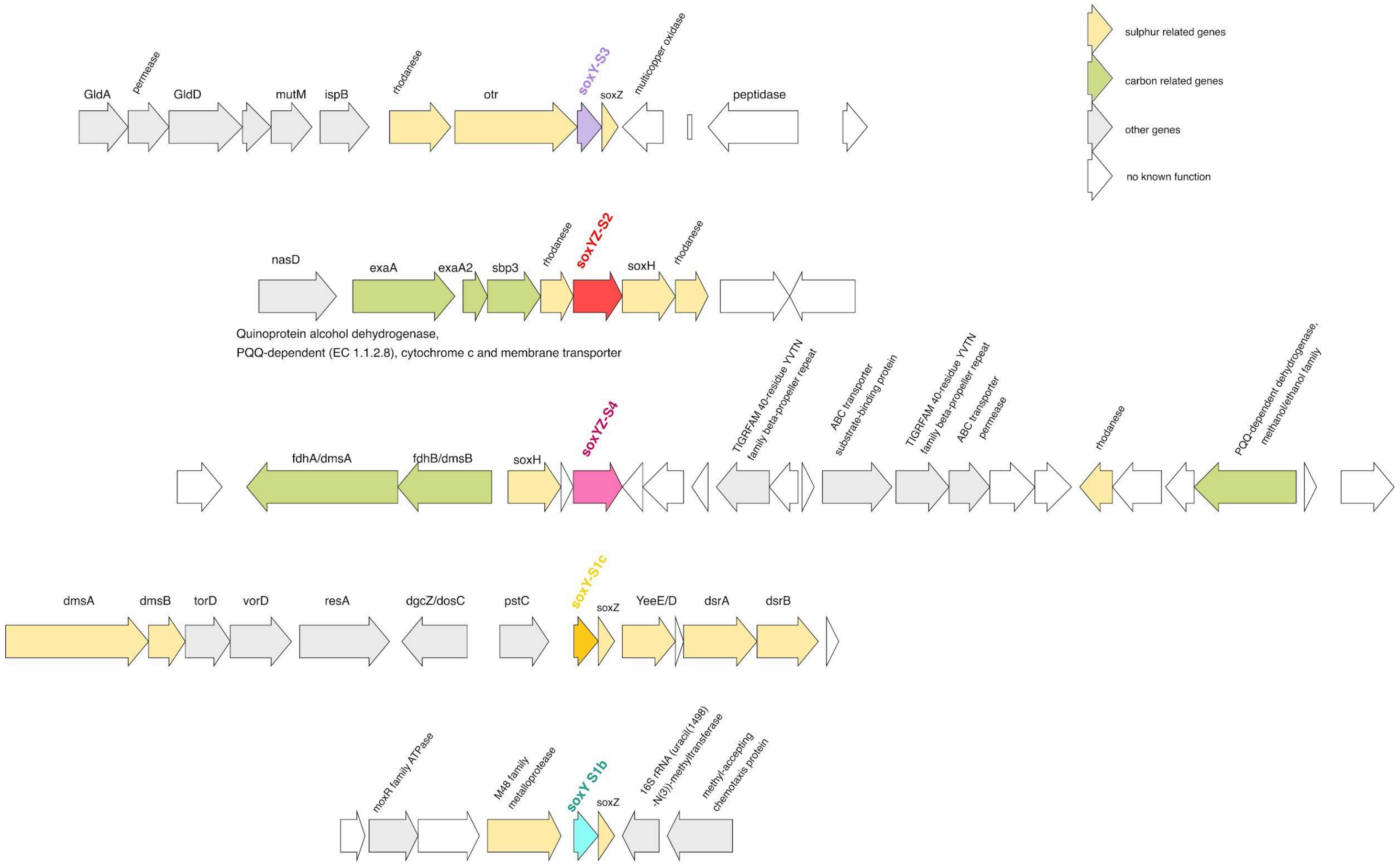

Figure S5: Genetic islands of divergent *soxY* signatures 1b, 1c, 2-4. Yellow coloring represents sulfur metabolism related genes (including: *dmsAB* - dimethyl sulfoxide reductase, YeeE/D - thiosulfate transporter, *dsrAB* - dissimilatory (bi)sulfite reductase, *soxH*; green - carbon related genes (ADH - quinohemoprotein alcohol dehydrogenases, ADH acc. - ADH accessory genes, *fdhAB* - formate dehydrogenase, *xoxF* - lanthanide-dependent methanol dehydrogenase); gray - other/multifunctional genes (*rhd* - rhodanese), white genes are of unknown functions.

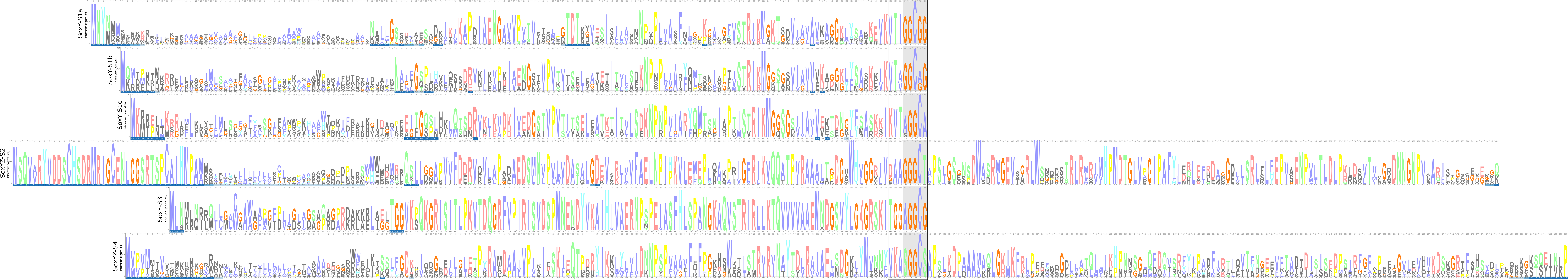
SoxY-S1a
SoxY-S1b
SoxY-S1c
SoxYZ-S2
SoxY-S3
SoxYZ-S4

Figure S6: Logo plots of all SoxY protein sequences according to their signature. The conserved "swinging arm" domain with the thiosulfate binding cysteine residue is marked by a grey box, and grey fill. Each logo was calculated based on alignment of symbiotic amino acid sequences according to the signature naming.

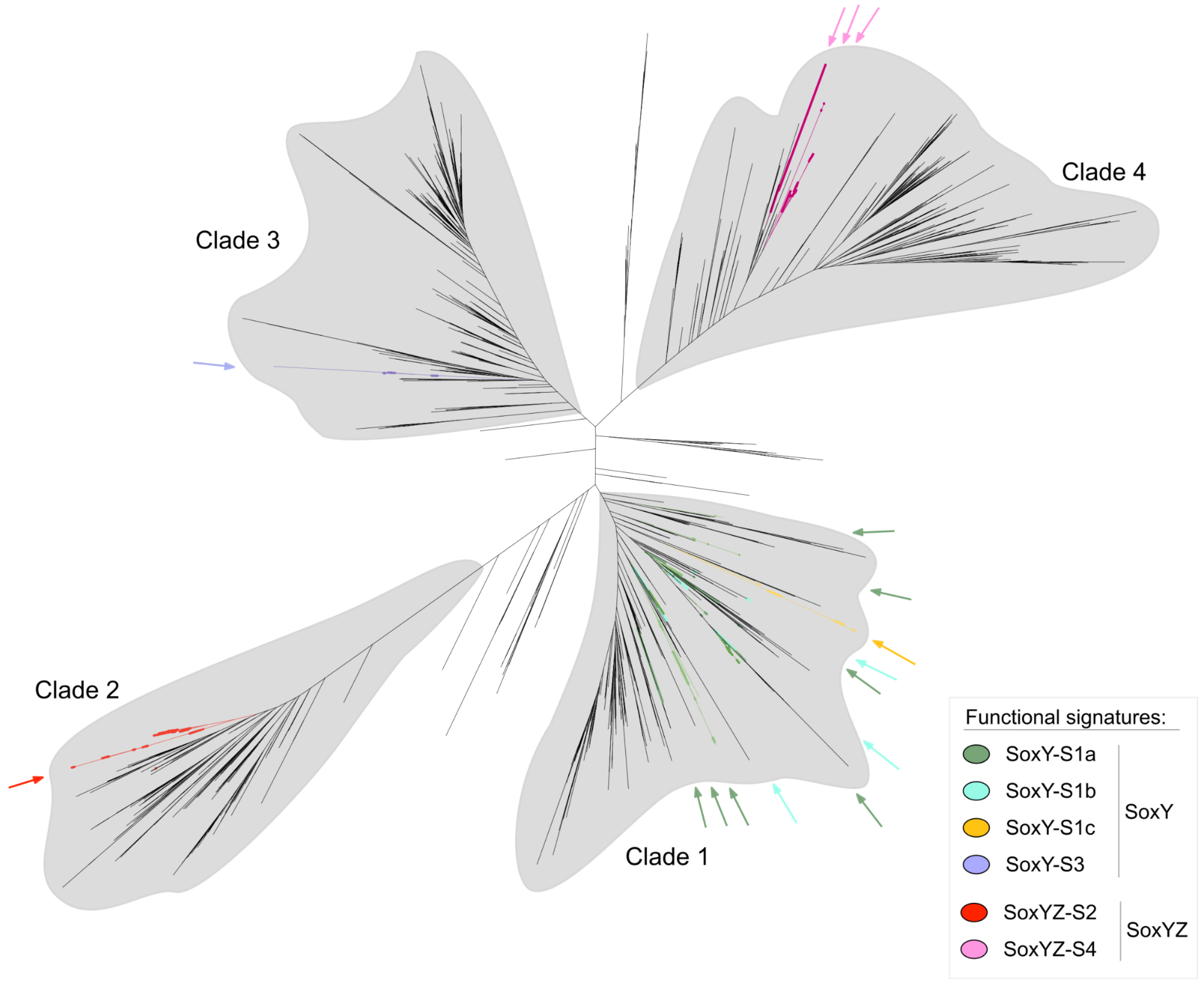

Figure S7: Maximum likelihood tree of SoxY proteins (1642 sequences total) from symbionts and free-living bacteria including sequences from NCBI and Pfam (RP55). The major clades are labeled according to their phylogeny. The SoxY sequence signature groups from symbiotic SOB were distinguished by mutations within the sulfur-binding "swinging arm" of the protein. The canonical SoxY sequence with its conserved sulfur-binding cysteine-110 (cys110) is labeled as SoxY-S1a. Arrows indicate the location of the symbiotic SoxY sequences on the tree.

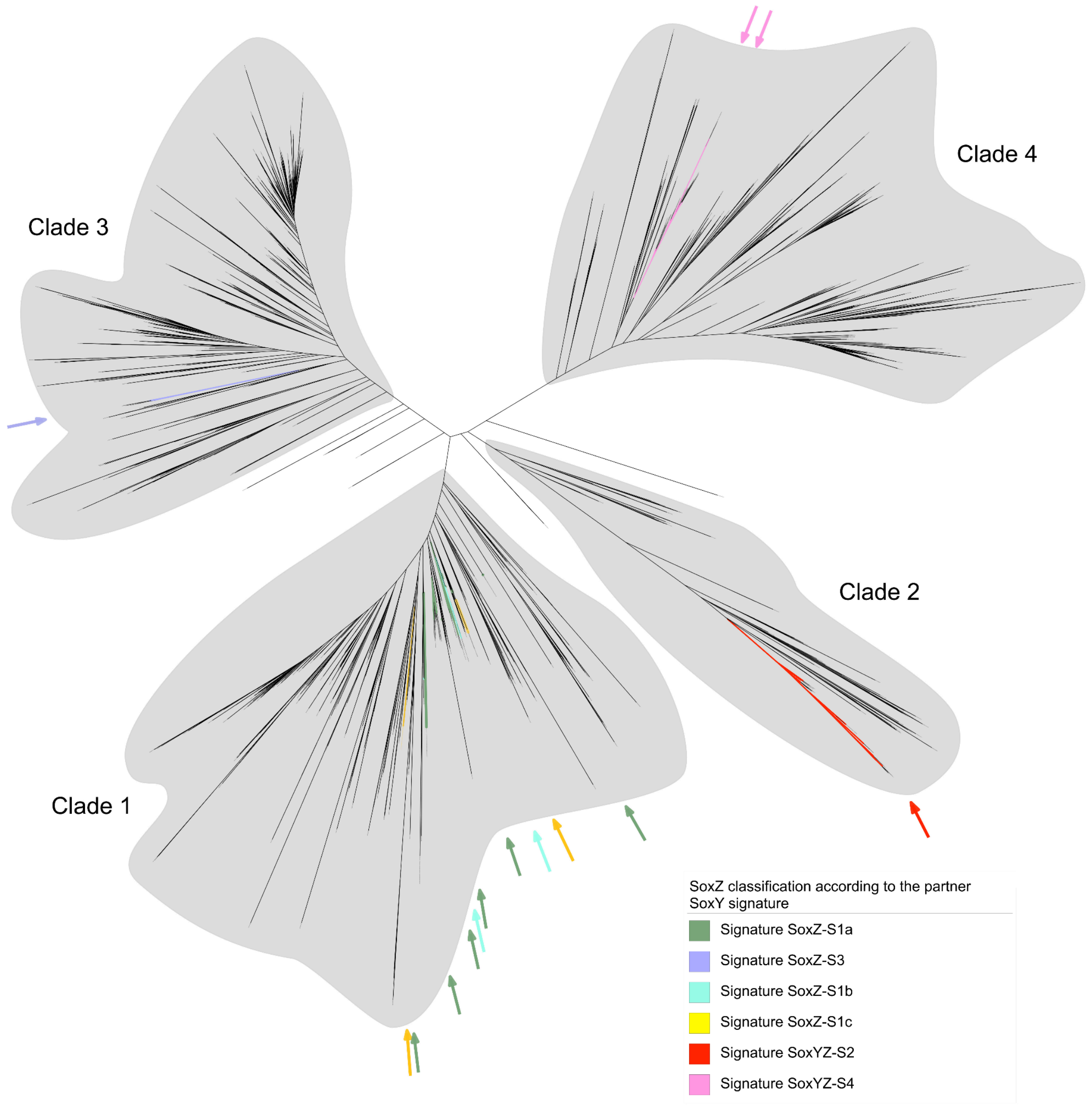

Figure S8: Maximum likelihood tree of SoxZ proteins (1693 sequences total) from symbionts and free-living bacteria including sequences from NCBI and Pfam (RP55). The major clades are labeled according to SoxY phylogeny. The SoxZ sequence signature groups from symbiotic SOB were distinguished according to the SoxY classification and mutations within the sulfur-binding “swinging arm” of the SoxY protein. Arrows indicate the location of the symbiotic SoxZ sequences on the tree.

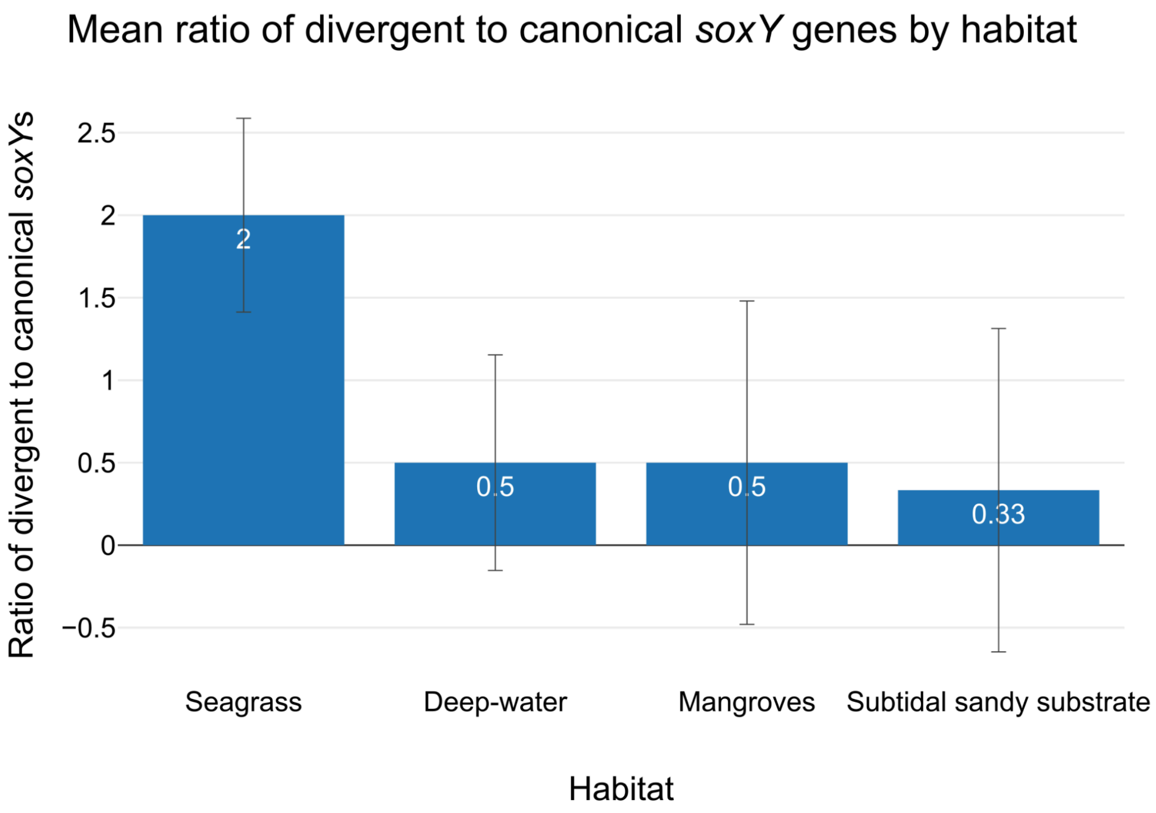

Figure S9: Mean ratio of the number of divergent *soxY* gene copies to canonical *soxY* gene copies within the same lucinid symbiont species according to the habitat. Only symbiont species represented by more than one MAG has been included in the analysis. Due to statistical requirements only habitats inhabited by more than one symbiont species has been considered.

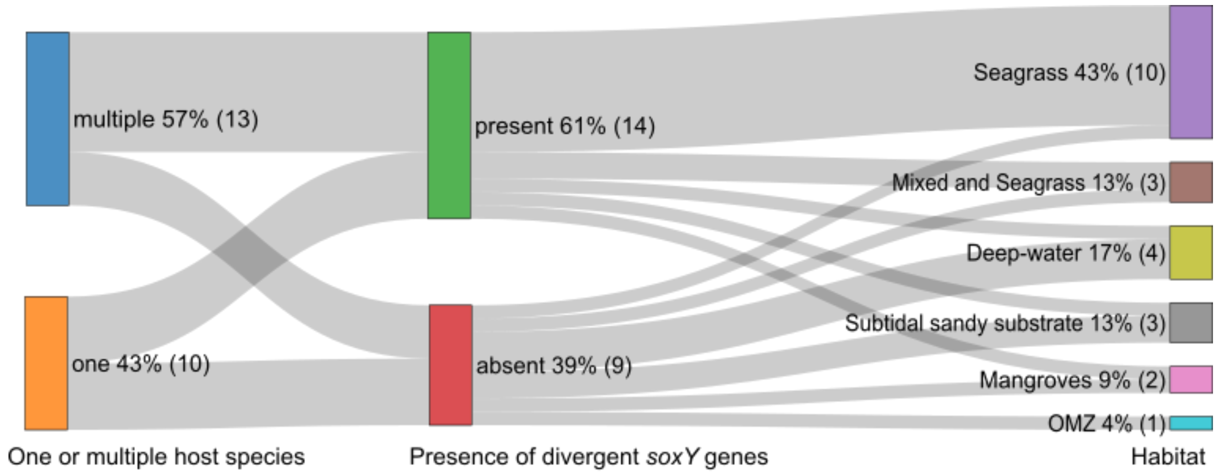

Figure S10: Relationship between number of host species, presence of divergent *soxY* gene and the habitat in lucinid symbioses as Sankey Diagram. Only symbiont species represented by more than one MAG has been included in this diagram.
