# Supplementary material for: SoxY gene family expansion underpins adaptation to diverse hosts and environments in symbiotic sulfide oxidizers
Source: mSystems. 2024 May 15;9(6):e01135-23. doi: 10.1128/msystems.01135-23 (PMC11237559; doi:10.1128/msystems.01135-23)
Supplement: Supplemental text — Supplemental methods and results. [file msystems.01135-23-s0002.docx]

**Supplementary methods:**

**DNA Extraction methods:**

Approximately 1 cm2 gill pieces were cut into 2 mm fragments and DNA was extracted using the Qiagen DNeasy Blood and Tissue kit (Cat. No. 69506; Qiagen, USA) or the animal tissue protocol from the Analytik Jena Innuprep DNA Mini Kit (Cat. No. 845-KS-1041250, Germany), following the manufacturers’ instructions with the following modifications: (1) Tissues were incubated in the lysis buffer solution provided in each kit until completely digested (2–20 h); (2) DNA was eluted in 50 µl of ultrapure water (Ref. 10977-015; Invitrogen, Life Technologies, USA). The extracted DNA was quantified using the Qubit dsDNA High Sensitivity Assay kit (Thermo Fisher Scientific, USA) and stored at −20 °C.

Trizol DNA/RNA extraction protocol (bead beating) adapted from F_071215 Invitrogen Trizol detailed protocol

Homogenization using bead beater (20min for ~ 4 samples)

1. Add gill sample sample to bead beater lysis tube
2. Add 990μl Trizol
3. Add 12.5μl Glycogen (20mg/mL)
4. Put tubes into bead beater for 10s, 4.0m/s
5. Incubate at RT for 5 min

Phase separation #1

1. Spin at max speed for 10 mins, 4°C
2. Transfer supernatant into new tube
3. Add 160μl BCP
4. Invert or shake for 15 s and incubate at RT for 15 mins
5. Spin at max speed for 15 mins, 4°C. 3 layers should be visible
6. Transfer colorless aqueous phase (RNA) into new eppie

Phase separation #2

1. Remove remaining aqueous phase
2. Add 0.5 initial-Trizol volume (500μl to 1ml) of back extraction buffer
3. Shake tubes vigorously by hand for 15 sec.
4. Incubate 10 min at RT
5. Phase separation: centrifuge samples 12 000 g (10 620 rpm*), 15 min, 4°C
6. Transfer the aqueous phase containing DNA to a new tube

DNA precipitation

1. Add 12.5μl Glycogen (20mg/mL)
2. Add 0.4 ml of isopropanol per 1 ml of Trizol used initially (400μl per 1ml trizol)
3. Mix by inversion and incubate at -80°C for 1 hour – overnight

DNA wash and elution

1. Spin 12000 x g (10 620 rpm*) for 5 min at 4-25°C and remove the supernatant
2. Add 1 ml of 0.1 M Sodium Citrate in 10% ethanol (pH 8.5) to the DNA pellet, mix by inversion, incubate for 30 min at room temperature
3. Centrifuge samples 4 000 g for 5 min 4°C, discard supernatant
4. Wash by adding 1 ml 75% EtOH, incubate for 15 min at room temperature
5. Centrifuge samples 4 000 g for 5 min 4°C to pellet the DNA
6. Discard the supernatant
7. Remove ethanol, re-spin a few seconds and remove remaining ethanol
8. Air dry the DNA pellet for 5-10 min, but do not allow the pellet to dry out
9. Add 70 μl of Nuclease-free water (pre-heat to 65°C), TE per 10 mg of tissue (final conc. should be 0.2-0.3 μg/μl) by slow and gentle pipetting.

Expected DNA yield: 3-7 μg
rpm values for Eppendorf microcentrifuge 5430R (FA-45-30-11)

**Genus-level classification of novel symbiont species**

The genus-level classification of two novel symbiont species, sister clades to *Ca*. Sedimenticola endophaicodes has been done by pairwise comparison using the Percentage Of Conserved Proteins calculation, as proposed by Qin, Xie et al. 2014 ((1); script: <https://figshare.com/articles/software/POCP_calculation_for_two_genomes/4577953/1> from Moose A. POCP calculation for two genomes. 2017), which utilizes blastp (BLAST+ 2.14.0) (2).

**Alignment and visualization of symbiotic SoxY signatures**

All symbiotic SoxY sequences have been classified according to their functional signature and aligned separately using hmmalign (3) (default settings) and the SoxY model retrieved from eggNOG database (4). The alignments have been visualized using [Skylign](http://www.biomedcentral.com/1471-2105/15/7/) (5) and Inkscape 1.3.2.

**Phylogenetic analysis of SoxZ sequences**

The SoxZ sequences neighboring SoxY were extracted using the SoxZ HMM model obtained from the eggNOG database (4) (accessed January 2022) and using the hmmsearch tool in HMMER v3.3.2 (3) with an e-value cutoff of 10^-5^ (based on the observed sequence identity of SoxY and SoxZ proteins). Further RP55 (PF08770) of SoxZ sequences has been obtained. The final set of 1705 full-length SoxZ sequences were aligned using hmmalign (3) (default settings including automatic trimming for poorly aligned regions and gaps) and the SoxZ model retrieved from eggNOG database (4). The alignment was visualized using MEGA X (6). A maximum likelihood tree was constructed using IQ-Tree multicore version 2.1.2 (7, 8) with the Q.pfam+G4 model (ModelFinder (9) was used to determine the best substitution model) and 1000 ultra-rapid bootstraps (UFBoot)(10). The final consensus tree was visualized and annotated using Interactive Tree Of Life (iTOL) v6.8 (11) as well as Inkscape 1.3.2.

**Statistical test and visualization of the relationship between the presence of divergent *soxY* and host species as well as habitat type**

The statistical test investigating the relationship between the presence of divergent *soxY* and host species as well as habitat type was non-parametric because the data was not normally distributed according to the Shapiro–Wilk test. Kruskal-Wallis Test was used for comparing two or more independent samples of equal or different sample sizes and a Dunn-Bonferroni test was used to compare the groups in pairs to find out which was significantly different. The relation of the ratio of divergent *soxY* genes to canonical *soxY* genes and association with one or multiple host species has been calculated using Mann-Whitney U test. All statistical calculations have been performed using DATAlab (12).

**Supplementary results:**

**Genus-level classification of two new *Sedimenticola* species**

The two new species, *Ca*. Sedimenticola endoloripinus and Sedimenticola3, clustered outside of the well described *Thiodiazostropha* genus and were neighboring clades to *Ca*. Sedimenticola endophacoides belonging to *Sedimenticola* genus*.* We assign these two new symbiont species likewise to *Sedimenticola* genus based on the phylogenetic tree structure and the pairwise percentage of conserved proteins shared among these newly described symbionts and *Ca*. S. endophacoides, which falls within the range of 59-66.5%. This range exceeds the 50% threshold proposed by Qin et al., 2014 (1) as the genus-level boundary.

**The potential co-evolution of the SoxY and SoxZ**

The SoxY as well as SoxZ sequences in their corresponding phylogenetic tree form four major clades suggesting similar evolutionary paths (Figure S7 and S8). The symbiotic SoxY and their corresponding SoxZ sequences were separated following the same trend with clade 1 containing SoxY-S1a-c and the corresponding SoxZ, clade 2 and clade 4 contained SoxYZ-S2 and SoxYZ-S4 fusion protein respectively and clade 3 contained symbiotic SoxY-S3 and corresponding SoxZ. It was not possible to calculate percentage concordance between the SoxY and SoxZ trees because the sequences in each alignment were obtained directly from the UniRef50 database and may not necessarily represent the full complement of either gene from their respective genomes.

**The relationship between the presence of divergent *soxY* and host species as well as habitat type**

The Kruskal-Wallis test showed that there was a significant difference (*p*=.027) in the ratio of divergent *soxY* genes and canonical *soxY* genes between the habitats. Despite the significant difference in the Kruskal-Wallis test, no pairwise habitat group comparison was significant in the Dunn-Bonferroni test; all adjusted p-values were greater than 0.05.

|  | Test Statistic | Std. Error | Std. Test Statistic | p | Adj. p |
| --- | --- | --- | --- | --- | --- |
| Seagrass - Subtidal sandy substrate | 8.6 | 3.91 | 2.2 | .028 | .167 |
| Seagrass - Deep-water | 7.64 | 3.49 | 2.19 | .029 | .171 |
| Seagrass - Mangroves | 7.77 | 4.64 | 1.68 | .094 | .563 |
| Subtidal sandy substrate - Deep-water | -0.96 | 4.66 | -0.21 | .837 | 1 |
| Subtidal sandy substrate - Mangroves | -0.83 | 5.57 | -0.15 | .881 | 1 |
| Deep-water - Mangroves | 0.13 | 5.29 | 0.02 | .981 | 1 |

While comparing the relation of the ratio of divergent *soxY* genes to canonical *soxY* genes and association with one or multiple host species, the “one host” group had lower values for the dependent variable ratio of divergent to canonical than the “multiple host” group. A Mann-Whitney U test was conducted to compare scores between “one host” and “multiple hosts”. For the given data a Mann-Whitney U-Test showed that the difference between “one host” and “multiple hosts” with respect to the dependent variable ratio of divergent to canonical was not statistically significant, U = 44, n1 = 9, n2 = 13 p = .357.

**Literature**:

1. Qin Q-L, Xie B-B, Zhang X-Y, Chen X-L, Zhou B-C, Zhou J, Oren A, Zhang Y-Z. 2014. A Proposed Genus Boundary for the Prokaryotes Based on Genomic Insights. J Bacteriol 196:2210–2215.

2. Mahram A, Herbordt MC. 2015. NCBI BLASTP on high-performance reconfigurable computing systems. ACM Transactions on Reconfigurable Technology and Systems (TRETS) 7:1–20.

3. Finn RD, Clements J, Eddy SR. 2011. HMMER web server: interactive sequence similarity searching. Nucleic Acids Research 39:W29–W37.

4. Huerta-Cepas J, Szklarczyk D, Heller D, Hernández-Plaza A, Forslund SK, Cook H, Mende DR, Letunic I, Rattei T, Jensen LJ, Von Mering C, Bork P. 2019. EggNOG 5.0: A hierarchical, functionally and phylogenetically annotated orthology resource based on 5090 organisms and 2502 viruses. Nucleic Acids Research 47:D309–D314.

5. Wheeler TJ, Clements J, Finn RD. 2014. Skylign: a tool for creating informative, interactive logos representing sequence alignments and profile hidden Markov models. BMC Bioinformatics 15:7.

6. Kumar S, Stecher G, Li M, Knyaz C, Tamura K. 2018. MEGA X: Molecular Evolutionary Genetics Analysis across Computing Platforms. Molecular Biology and Evolution 35:1547–1549.

7. Nguyen L-T, Schmidt HA, von Haeseler A, Minh BQ. 2015. IQ-TREE: A Fast and Effective Stochastic Algorithm for Estimating Maximum-Likelihood Phylogenies. Molecular Biology and Evolution 32:268–274.

8. Minh BQ, Schmidt HA, Chernomor O, Schrempf D, Woodhams MD, Von Haeseler A, Lanfear R, Teeling E. 2020. IQ-TREE 2: New Models and Efficient Methods for Phylogenetic Inference in the Genomic Era. Molecular Biology and Evolution 37:1530–1534.

9. Kalyaanamoorthy S, Minh BQ, Wong TKF, von Haeseler A, Jermiin LS. 2017. ModelFinder: fast model selection for accurate phylogenetic estimates. Nature Methods 14:587–589.

10. Hoang DT, Chernomor O, von Haeseler A, Minh BQ, Vinh LS. 2018. UFBoot2: Improving the Ultrafast Bootstrap Approximation. Molecular Biology and Evolution 35:518–522.

11. Letunic I, Bork P. 2021. Interactive tree of life (iTOL) v5: An online tool for phylogenetic tree display and annotation. Nucleic Acids Research 49:W293–W296.

12. DATAtab Team 2024. DATAtab: Online Statistics Calculator. DATAtab e.U. Graz, Austria. URL <https://datatab.net>
